# Spin-multiplexed point spread function engineering via dielectric metasurface for simultaneous optical differentiation and high-resolution imaging

**DOI:** 10.1038/s41377-026-02229-1

**Published:** 2026-07-15

**Authors:** Niu Liu, Zhelin Lin, Zhenyu Xing, Yuhui Hu, Yuxuan Liao, Amit Agrawal, Xinliang Zhang, Cheng Zhang

**Affiliations:** 1 School of Optical and Electronic Information & Wuhan National Laboratory for Optoelectronics, Huazhong University of Science and Technology, Wuhan, China; 2 Department of Engineering, University of Cambridge, Cambridge, UK; 3 Kyung Hee University, Seoul, Korea

**Keywords:** Metamaterials, Nanophotonics and plasmonics

## Abstract

In the era of artificial intelligence and machine learning, all-optical computing has garnered renewed attention for its intrinsic advantages in processing speed, energy efficiency, and parallelism over electronic systems. A key operation in such architecture is optical differentiation, which enables edge detection and feature extraction from target scenes. Metasurfaces—capable of controlling the amplitude, phase, and polarization of light at the nanoscale—have emerged as promising platforms for implementing compact optical differentiators. However, existing designs typically rely on auxiliary imaging optics to capture the processed outputs and are often limited in spatial resolution or differentiation order. Here, we introduce a new class of metasurface optical differentiators that simultaneously achieve arbitrary-order optical differentiation and high-resolution imaging in a standalone single-layer configuration, eliminating the need for additional optics. By precisely engineering multiple complex-valued point spread functions via spin multiplexing, our design enables on-demand, arbitrary-order differentiation directly across the light field and overcomes long-standing constraints in spatial resolution and operational flexibility. We experimentally demonstrate two devices capable of performing 0^th^/1^st^-order and 2^nd^/3^rd^-order differentiation with spatial resolution up to 228.0 lp/mm (line width of 2.19 μm). Their performance is further validated across diverse scenarios, including high-intensity illumination and real-time live cell imaging. Our results demonstrate that through rigorous point spread function engineering, metasurfaces offer a transformative platform for integrated, high-performance all-optical computing systems with broad potential in biological imaging, information processing, and material characterization.

## Abstract (web summary)

Through spin-multiplexed point spread function engineering, this work demonstrates single-layer metasurface optical differentiators that simultaneously achieve high-resolution imaging and on-demand, arbitrary-order differentiation without additional optics.

## Graphical abstract

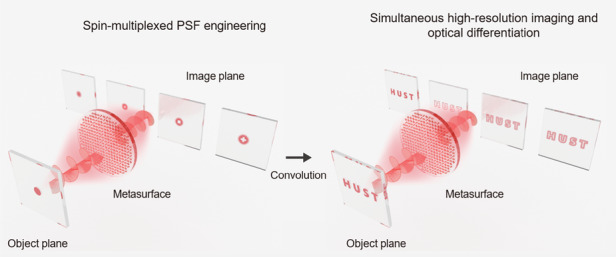

## Introduction

The performance of large language models, image recognition algorithms, or any other machine learning (ML) paradigms that underpin modern artificial intelligence (AI) systems fundamentally relies on efficient computation over massive training datasets. While conventional electronic computing systems have remarkably kept up with the unprecedented growth of AI through rapid advances in semiconductor technology^1,2^ and hardware architectures^3,4^, challenges remain in further reducing power consumption and accelerating processing speed^5^. All-optical computing, with its inherent advantages of low power consumption, fast processing speed, high throughput, and parallel computation capability, has emerged as a promising complimentary approach to address these challenges^6–11^. All-optical differentiator, which performs differentiation operation over target scenes through light propagation, forms the most critical component of optical computing system and has been widely used in image processing and signal analysis. Over recent years, metasurfaces – capable of precisely controlling the phase, amplitude, and polarization of incident light at the nanoscale by engineering the shape, size, and arrangement of their constituent nanostructures^12–29^ – have unlocked functionalities far beyond those of conventional optical elements and have emerged as a promising platform for implementing compact all-optical differentiators^30–37^.

Currently, there are two primary methods for implementing a metasurface-based optical differentiator: the 4-*f* system approach and the Green’s function (GF) approach. In the 4-*f* system approach, the desired transfer function for a target differentiation operation is realized by positioning a metasurface, acting as a spatial-frequency filter, at the Fourier plane of a 4-*f* system^38–43^. Although the metasurface-based 4-*f* system approach offers adequate modulation precision, it still requires additional lenses and physical space to perform the necessary Fourier and inverse Fourier transforms, making the optical system bulky. Also, the need for precise positioning of both the lens pair and the metasurface makes the system susceptible to alignment errors. In contrast, the GF approach achieves the desired transfer function by leveraging the nonlocality offered by metasurfaces, enabling differentiation to be performed using just a single metasurface without the need for the 4-*f* system^44–46^. However, since it is challenging to design a metasurface that offers ideal phase and amplitude modulation over the entire spatial-frequency space, the GF method necessitates approximating the phase and amplitude responses over a limited angular range (spatial-frequency range). Such constraint limits the achievable numerical aperture (i.e., spatial resolution) and restricts the variety of differentiations that can be performed, including the differentiation order and direction. Furthermore, this approach inherently lacks imaging capability, necessitating auxiliary imaging optics to capture the computed results. It is worth noting that in both these approaches, the demonstrated differentiation operations have typically been limited to low orders (primarily 1^st^- and 2^nd^- order). Higher-order differentiation, as required for advanced ML operations like differential equation solving and Taylor expansion operation, has recently been achieved by leveraging the nonlocal response of dielectric multilayer structures^47,48^. However, similar to the GF approach, these multilayer structures lack inherent imaging functionality, again necessitating the use of additional optics. Explorations in constructing spiral metalenses have offered new possibilities for all-optical differentiation. For instance, by incorporating hyperbolic phase and spiral phase with a topological charge of +1 at the metasurface plane, 1^st^-order differentiation can be implemented without relying on the 4-*f* architecture or any additional imaging optics^49–51^. Moving towards higher-order differentiation, the feasibility of employing Bessel vortex phase modulation has recently been explored^52^. In this approach, the asymptotic behavior of the Bessel function with near-zero variable is used to achieve differentiation of different orders, which at the same time, sets constraints on the applicable spatial-frequency range (i.e., achieved spatial resolution). Therefore, it is apparent that integrating high-resolution imaging with on-demand, arbitrary-order differentiation on a robust, low-size, weight, and power (SWaP) platform is critical for the advancement of all-optical computing systems, yet remains an outstanding challenge that warrants further investigation.

In this work, we demonstrate a new class of metasurface differentiators that leverage point spread function (PSF) engineering to seamlessly integrate high-resolution optical imaging with arbitrary-order optical differentiation in an ultracompact, alignment-free platform. Unlike previous embodiments that either require Fourier transform lens pair or supplementary imaging optics, our design achieves simultaneous multifunctionality all with one single-layer metasurface: it performs spin-multiplexed differentiation (e.g., 0^th^/1^st^- and 2^nd^/3^rd^-order differentiation) directly on target objects while simultaneously allowing high-resolution imaging. We achieve this functionality through rigorous PSF engineering of the metasurface, designed to provide independent and arbitrary modulation of the amplitude and phase of two orthogonal circularly polarized incident light states. By tailoring the complex-valued PSFs of the metasurface for each incident polarization state, the device enables switchable, multi-order differentiation of the image scene, generating the corresponding high-resolution, crosstalk-free differentiation results directly in the formed images without requiring additional imaging optics or device alignment. We implement two types of metasurface differentiators, each capable of performing 0^th^/1^st^-order and 2^nd^/3^rd^-order spin-multiplexed differentiation. The devices operate over a broad wavelength range spanning from yellow to near-infrared, and achieve fine spatial resolution up to 228.0 lp/mm (corresponding to a line width of 2.19 μm). In contrast to conventional image processing systems where the intensity information of a target scene is first captured by an imaging system and the resulting images are subsequently processed by digital methods, our all-optical differentiator provides enhanced details by performing differentiation directly over the light field, enabling instantaneous multi-dimensional light field data acquisition and processing. We demonstrate the robustness of our architecture under high intensity illumination and further validate its real-time imaging capability by observing live Euglena cells. We believe this work provides a realistic path towards realization of ultracompact all-optical computing devices with potential applications in biological imaging, material characterization, advanced information processing, and beyond.

## Results

### Working principle of the PSF-engineered metasurface differentiator

**Fig. 1:**
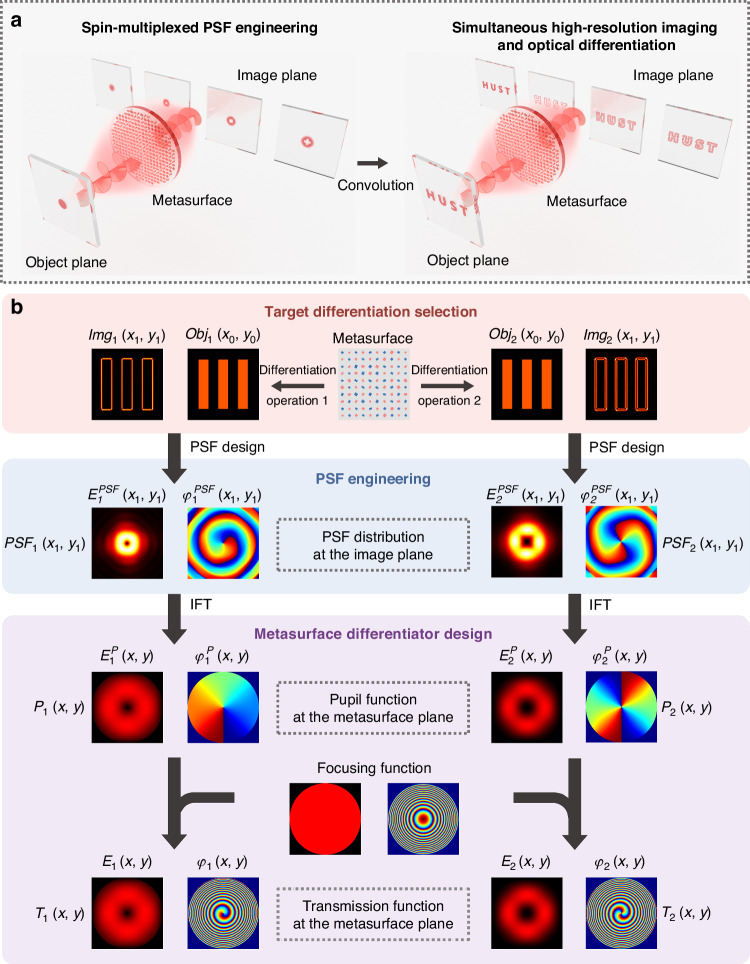
Conceptual illustration of the PSF-engineered metasurface differentiator. **a** Schematic representation of the proposed PSF-engineered metasurface differentiator. By precisely engineering its PSF, the device allows simultaneous execution of both high-resolution imaging and optical differentiation. **b** Flow chart illustrating the design process of the spin-multiplexed PSF-engineered metasurface differentiator, whose key steps include target differentiation selection, PSF engineering, and metasurface differentiator design. To realize two independent target differentiations, two distinct complex-valued PSFs are engineered for the single-layer spin-multiplexed metasurface. By applying inverse Fourier transform on the engineered PSFs, the target pupil functions and transmission functions at the metasurface plane are further derived

Figure 1a illustrates the schematic of the PSF-engineered metasurface differentiator. By precisely engineering its spin-multiplexed PSFs with complex value distributions, the device allows simultaneous execution of high-resolution imaging and optical differentiation, and generates two independent images of multiple differentiation orders depending on the spin state of the incident light. Under left-handed circularly polarized (LCP) illumination, the metasurface performs one order of differentiation and generates an output image; under right-handed circularly polarized (RCP) illumination, the metasurface carries out another independent order of differentiation over the same field of view and generates another output image at the same image plane.

In a single-lens imaging system, an output image is produced by convoluting the input object with PSF of the imaging system, where the PSF is determined by the lens’ pupil function. By engineering the system’s complex-valued PSF through modification of the associated pupil function, different output images with multiple differentiation orders can be produced. In this work, we utilize a spin-multiplexed dielectric metasurface which provides two independent PSFs, each for one of the two orthogonal circularly polarized light states, to implement multiplexed differentiation over a target object. A flowchart detailing the metasurface design process is illustrated in Fig. 1b. The output light field distribution at the image plane, ${{Img}}_{\mathrm{1,2}}({x}_{1},{y}_{1})$, is derived by convoluting the input light field at the object plane, ${{Obj}}_{\mathrm{1,2}}({x}_{0},{y}_{0})$, with the metasurface's PSF, ${PS}{F}_{\mathrm{1,2}}({x}_{1},{y}_{1})$.

1 $${{Img}}_{1,2}\left({x}_{1},{y}_{1}\right)={\iint }_{-\infty }^{\infty }{O{bj}}_{1,2}\left({x}_{0},{y}_{0}\right){PS}{F}_{1,2}\left({x}_{1}-{x}_{0},{y}_{1}-{y}_{0}\right)d{x}_{0}d{y}_{0}$$

where $({x}_{0},{y}_{0})$ and $({x}_{1},{y}_{1})$ are the two-dimensional (2D) coordinates of the object and the image plane, respectively. The subscripts 1 and 2 correspond to the target differentiation operation 1 and 2 under LCP and RCP illumination, respectively. ${P{SF}}_{\mathrm{1,2}}({x}_{1},{y}_{1})={E}_{\mathrm{1,2}}^{{PSF}}({x}_{1},{y}_{1})\exp [i{\varphi }_{\mathrm{1,2}}^{{PSF}}({x}_{1},{y}_{1})]$ is the PSF with amplitude ${E}_{\mathrm{1,2}}^{{PSF}}({x}_{1},{y}_{1})$ and phase ${\varphi }_{\mathrm{1,2}}^{{PSF}}({x}_{1},{y}_{1})$. Equation (1) indicates that various output imaging results can be generated by selecting an appropriate PSF. For example, an Airy-disk-shaped PSF leads to conventional bright-field imaging^53^, while a donut-shaped PSF leads to edge-enhanced imaging^54^. The complex-valued PSF generated by the metasurface can be obtained via Fourier transform of the pupil function at the metasurface plane:

2 $${PS}{F}_{1,2}\left({x}_{1},{y}_{1}\right)\propto F\left\{{P}_{1,2}\left(x,y\right)\right\}{|}_{u=\frac{{x}_{1}}{{\lambda }_{0}{f}_{0}},v=\frac{{y}_{1}}{{\lambda }_{0}{f}_{0}}}$$

where $(x,y)$ is the 2D coordinate at the metasurface plane; $(u,v)$ is the spatial frequency of the metasurface-modulated light field along the *x-* and *y*-axis, respectively; $F$ denotes the Fourier transform operation; ${f}_{0}$ is the focal length of the metasurface; and ${\lambda }_{0}$ is the free-space wavelength. ${P}_{\mathrm{1,2}}(x,y)={E}_{\mathrm{1,2}}^{P}(x,y)\exp [i{\varphi }_{\mathrm{1,2}}^{P}(x,y)]$ is the pupil function with amplitude ${E}_{\mathrm{1,2}}^{P}(x,y)$ and phase ${\varphi }_{\mathrm{1,2}}^{P}(x,y)$. Detailed derivation of ${P}_{\mathrm{1,2}}(x,y)$ is presented in Section S1, Supplementary Information.

Here, to achieve differentiation of multiple orders through PSF engineering requires implementing multiple independent pupil functions on the metasurface. The design process is inspired by conventional digital image processing, where convolution kernels are employed to realize differentiation of various orders. For the 0^th^-order differentiation, the target complex-valued pupil function is derived by applying inverse Fourier transform (IFT) on the Gaussian convolution kernel ($${H}_{0}=\frac{1}{16}\left[\begin{array}{ccc}1 & 2 & 1\\ 2 & 4 & 2\\ 1 & 2 & 1\end{array}\right]$$). For the 1^st^-order differentiation, we first utilize the horizontal and vertical Sobel convolution kernels to construct an isotropic 1^st^-order differential kernel ($${H}_{1}={H}_{x}+i* {H}_{y}=\left[\begin{array}{ccc}-1 & 0 & 1\\ -2 & 0 & 2\\ -1 & 0 & 1\end{array}\right]+i* \left[\begin{array}{ccc}-1 & -2 & -1\\ 0 & 0 & 0\\ 1 & 2 & 1\end{array}\right]$$), and then apply IFT on the constructed kernel to obtain the target complex-valued pupil function. For higher-order differentiation, which can be considered as successive applications of 1^st^-order differentiation, the target pupil function is obtained by recursively multiplying the results of the 1^st^-order differentiation, where the number of multiplications correspond to the desired differentiation order. It is worth noting that while the obtained phase profiles appear to be vortex-like, they are instead custom-optimized results for specific differentiation orders. Our approach enables on-demand, arbitrary-order differentiation, demonstrating capabilities that are not feasible with the vortex-phase approach.

The metasurface implemented in this work functions as both a computing and an imaging element, with its computational capability enhanced by the spin-multiplexed response. Here, computing is realized by imparting independent and arbitrary phase and amplitude modulation to a pair of circularly polarized incident light, thereby generating the desired pupil function for each polarization state at the metasurface plane. In parallel, imaging is achieved by imparting a hyperbolic focusing phase to both the LCP and RCP light incident on the metasurface. This ensures that the device generates computed imaging outputs over the same field of view, but under two distinct differentiation modes. Therefore, the overall light-field modulation (i.e., the transmission function) implemented via the metasurface is obtained by combining the pupil function and the focusing function:

3 $$\begin{array}{l} {T}_{1,2}\left(x,y\right)={P}_{1,2}\left(x,y\right)\cdot H\left(x,y\right)={E}_{1,2}^{P}(x,y)exp\left[i{\varphi }_{1,2}^{P}(x,y)\right]\cdot exp\left[i{\varphi }_{{lens}}(x,y)\right] \\ ={E}_{1,2}\left(x,y\right)exp\left[i{\varphi }_{1,2}\left(x,y\right)\right]\end{array}$$

where ${T}_{\mathrm{1,2}}\left(x,y\right)$ is the target transmission function of the metasurface; ${P}_{\mathrm{1,2}}\left(x,y\right)$ is the pupil function at the metasurface plane; $H\left(x,y\right)$ is the focusing function; ${\varphi }_{{lens}}=-\frac{2\pi }{{\lambda }_{0}}\left(\sqrt{{{f}_{0}}^{2}+{x}^{2}+{y}^{2}}-{f}_{0}\right)$ is the hyperbolic focusing phase; ${E}_{\mathrm{1,2}}\left(x,y\right)$ and ${\varphi }_{\mathrm{1,2}}\left(x,y\right)$ are the target amplitude and phase distribution implemented by the metasurface, respectively. Once the desired spin-multiplexed transmission function is determined, the metasurface can then be designed to provide simultaneous phase and amplitude modulation over a pair of LCP and RCP incident light and generate two independent complex-valued PSFs, enabling direct-imaging differentiation of multiple orders over the target object. A detailed description of the pupil function, focusing function, and transmission function for the designed differentiator is given in Section S2, Supplementary Information.

### Device design and fabrication

**Fig. 2:**
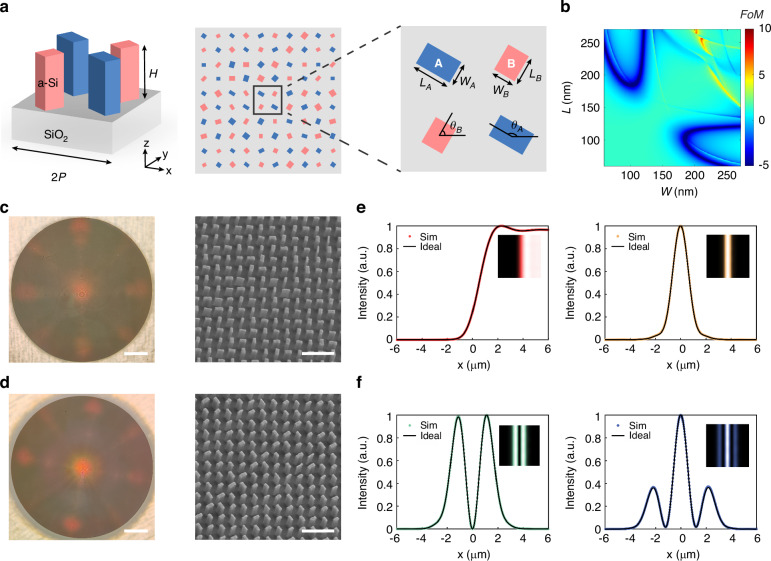
Design and fabrication of the spin-multiplexed metasurface differentiator. **a** Schematic representation of the metasurface super cell, comprising a pair of staggered twin amorphous silicon (a-Si) meta-atoms (respectively designated as A and B) on fused silica substrate. Both kinds of the a-Si meta-atoms have a rectangular-shaped cross-section, height of $H=530\,{nm}$, and lattice period of $P=350\,{nm}$. The cross-sectional dimensions and in-plane rotation angle for the two kinds of meta-atoms are set as $({W}_{A,B},{L}_{A,B})$ and ${\theta }_{A,B}$, respectively. **b** Half-waveplate figure-of-merit (*FoM*) versus a-Si meta-atom in-plane dimensions $(W,L)$, where the blue-color regions correspond to various combinations of $W$ and $L$ that satisfy the half-waveplate-like operation. **c** Left panel: Optical microscopy image of the fabricated 3-mm-diameter metasurface MS1. Scale bar: 0.5 mm. Right panel: SEM image of details of the fabricated a-Si nanopillars in MS1. View angle: 25°. Scale bar: 1 μm. **d** Left panel: Optical microscopy image of the fabricated 3-mm-diameter metasurface MS2. Scale bar: 0.5 mm. Right panel: SEM image of details of the fabricated a-Si nanopillars in MS2. View angle: 25°. Scale bar: 1 μm. **e** Simulated 0^th^-order (left panel) and 1^st^-order (right panel) differentiation responses of MS1 to a unit step function. **f** Simulated 2^nd^-order (left panel) and 3^rd^-order (right panel) differentiation responses of MS2 to a unit step function

To implement the target transmission function, ${T}_{\mathrm{1,2}}\left(x,y\right)$, we employ a super-cell strategy to construct the metasurface, where each super-cell comprises a pair of staggered twin dielectric meta-atoms (designated as A and B, Fig. 2a). Each meta-atom in the super-cell is engineered to function as a half-waveplate at the target operational wavelength, providing anisotropic phase modulation for LCP and RCP incident light. By leveraging interference between the transmitted light fields passing through each of the staggered twin meta-atoms, the metasurface can impose two independent phase and amplitude modulation profiles over a pair of circularly polarized light. For the target field distribution, ${E}_{1}(x,y)\exp \left[i{\varphi }_{1}\left(x,y\right)\right]$ for LCP illumination and ${E}_{2}(x,y)\exp \left[i{\varphi }_{2}\left(x,y\right)\right]$ for RCP illumination, the phase modulation ${\varphi }_{A,B}^{x}(x,y)$ and ${\varphi }_{A,B}^{y}(x,y)$ respectively for *x* and *y* polarized light and the rotation angle ${\theta }_{A,B}(x,y)$ of the meta-atoms A and B are given by:

4 $$\left\{\begin{array}{l}{\varphi }_{A,B}^{x}\left(x,y\right)=\frac{1}{2}\left({\varphi }_{A,B}^{+}\left(x,y\right)+{\varphi }_{A,B}^{-}\left(x,y\right)\right)\\ {\varphi }_{A,B}^{y}\left(x,y\right)=\frac{1}{2}\left({\varphi }_{A,B}^{+}\left(x,y\right)+{\varphi }_{A,B}^{-}\left(x,y\right)\right)+\pi \\ {\theta }_{A,B}\left(x,y\right)=\frac{1}{4}\left({\varphi }_{A,B}^{+}\left(x,y\right)-{\varphi }_{A,B}^{-}\left(x,y\right)\right)\end{array}\right.$$

Here, ${\varphi }_{A,B}^{x}(x,y)$ and ${\varphi }_{A,B}^{y}(x,y)$ differs by a value of *π*, consistent with the earlier statement that each meta-atom acts as a half-waveplate at the target operational wavelength of the metasurface. ${\varphi }_{A,B}^{+}\left(x,y\right)$ and ${\varphi }_{A,B}^{-}\left(x,y\right)$ are intermediate variables satisfying:

5 $$\left\{\begin{array}{c}\begin{array}{c}{\varphi }_{A}^{+}\left(x,y\right)={\cos }^{-1}\left({E}_{1}\left(x,y\right)\right)+{\varphi }_{1}\left(x,y\right)\\ {\varphi }_{B}^{+}\left(x,y\right)={\varphi }_{1}\left(x,y\right)-{\cos }^{-1}\left({E}_{1}\left(x,y\right)\right)\end{array}\\ {\varphi }_{A}^{-}\left(x,y\right)={\cos }^{-1}\left({E}_{2}\left(x,y\right)\right)+{\varphi }_{2}\left(x,y\right)\\ {\varphi }_{B}^{-}\left(x,y\right)={\varphi }_{2}\left(x,y\right)-{\cos }^{-1}\left({E}_{2}\left(x,y\right)\right)\end{array}\right.$$

Further details of the derivation process are presented in Section S3, Supplementary Information. Based on the analysis presented above, a unique pair of twin meta-atoms A and B can be selected to form the super-cell at each location $(x,y)$ at the metasurface plane, where their geometric parameters are adjusted spatially to satisfy the required anisotropic phase modulation, ${\varphi }_{A,B}^{x}(x,y)$ and ${\varphi }_{A,B}^{y}(x,y)$, and their rotation angles are respectively set as ${\theta }_{A}(x,y)$ and ${\theta }_{B}(x,y)$.

In this work, the device’s operational free-space wavelength is set to be ${\lambda }_{0}$ = 671 nm. We choose amorphous silicon (a-Si), which exhibits high refractive index and low optical loss at this wavelength, as the constituent material of the meta-atoms. The shape of the meta-atom is chosen to be a nanopillar of rectangular in-plane cross-section (major and minor axis length, $W$ and $L$, respectively), which provides anisotropic phase modulations for *x*- and *y*-polarized light. The height (*H*) and lattice period (*P*) of the nanopillar are set to be 530 nm and 350 nm, respectively. We then compute the phase shift and transmittance (${\varphi }_{x}$, ${T}_{x}$) and (${\varphi }_{y}$, ${T}_{y}$) at ${\lambda }_{0}$ = 671 nm for light polarized along the major and minor axis of the rectangular Si nanopillars with varying cross-sectional dimensions $(W,L)$ using finite-difference time-domain (FDTD) simulations (Section S4, Supplementary Information). In simulations, we iteratively vary $W$ and $L$ to identify orthogonal principal axis combinations that simultaneously lead to |${\varphi }_{x}-{\varphi }_{y}$|$\approx \pi$ and ${T}_{x}{\approx T}_{y}$ (in other words, half-waveplate operation). To facilitate the search of optimal nanopillar half-waveplates, a figure-of-merit (*FoM*) function is defined as ${FoM} = {\log_{10}}\left(\left|\frac{T_x}{T_y}\exp(i(\varphi_x-\varphi_y))-\exp(i\pi)\right|\right)$ and displayed in Fig. 2b, where regions of low-*FoM* values correspond to combinations of $W$ and $L$ that satisfy the target half-waveplate operation. Through such process, a meta-atom library is constructed. For a given metasurface design, the required anisotropic phase modulations, ${\varphi }_{A,B}^{x}(x,y)$ and ${\varphi }_{A,B}^{y}(x,y)$, of the twin meta-atoms A and B, can be calculated from Eqs. (4) and (5). The geometric parameters of the meta-atoms are then determined by spatially mapping the required phase values to the pre-constructed meta-atom library. The in-plane dimensions and *FoM* values of the selected meta-atoms are provided in Section S5, Supplementary Information. For experimental demonstration, we choose to implement two metasurface devices: one performing 0^th^/1^st^-order differentiation (labeled MS1) and the other performing 2^nd^/3^rd^-order differentiation (labeled MS2). Each device has a diameter of *D* = 3.000 mm and numerical aperture of *NA* = 0.3, corresponding to a focal length of ${f}_{0}$ = 4.770 mm. To further assess the robustness of the metasurface design, we numerically simulate devices with random cross-sectional width and length variations of ±5 nm, ±10 nm, and ±20 nm in the meta-atoms. The resulting devices exhibit robust performance, maintaining high PSF fidelity and imaging quality (Section S6, Supplementary Information).

The metasurface fabrication process includes a-Si film deposition using plasma-enhanced chemical vapor deposition (PECVD), electron beam lithography (EBL), chromium (Cr) etching mask deposition and lift-off, and inductively coupled plasma reactive ion etching (ICP-RIE). Detailed fabrication process is described in Materials and Methods. The scanning electron microscopy (SEM) images and optical micrographs of the fabricated devices MS1 and MS2 are presented in Fig. 2c–d, respectively. The simulated multi-order differentiation is validated through unit step function analysis (Fig. 2e–f). MS1 demonstrates two distinct operational modes: (*i*) bright-field imaging via 0^th^-order differentiation and (*ii*) single-edge extraction through 1^st^-order differentiation. MS2 further extends this paradigm, generating double-edge response (2^nd^-order differentiation) and triple-edge response (3^rd^-order differentiation) at step transitions – behaviors that align precisely with the n^th^-order differentiation operator implemented by the engineered PSF.

### Device characterization

**Fig. 3:**
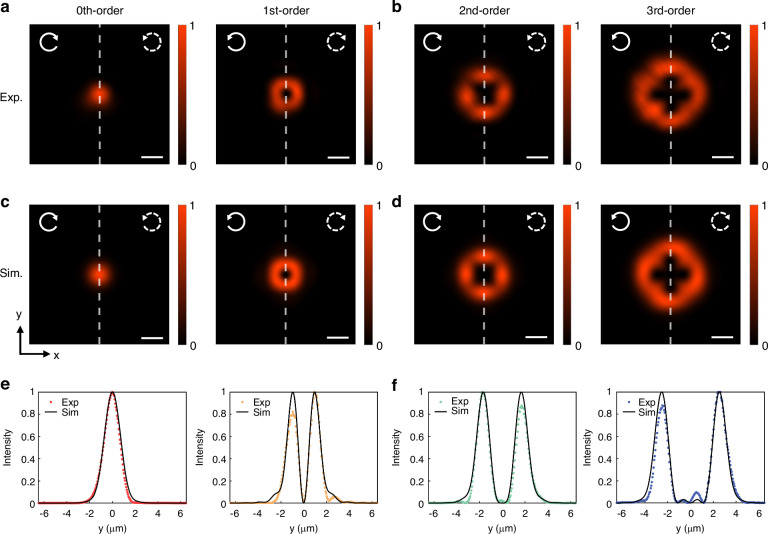
PSF characterization of the fabricated spin-multiplexed metasurface differentiators. **a–d** Measured PSF intensity profiles (normalized) of the device MS1 (**a**) and MS2 (**b**) under LCP (left panel) and RCP (right panel) illumination at free-space wavelength of 671 nm. Simulated PSF intensity profiles (normalized) of the device MS1 (**c**) and MS2 (**d**) under LCP (left panel) and RCP (right panel) illumination at free-space wavelength of 671 nm. Scale bars for Fig. 3a–d: 2 μm. The solid and dash arrows in the top part of each figure represent the polarization states of the incident light and transmitted light, respectively. The clockwise arrow denotes LCP, while the counterclockwise arrow denotes RCP. **e**–**f** Cross-sectional plots (dashed lines) of the measured PSFs along the vertical direction in Fig. 3a–b, respectively. The associated theoretically predicted curves are plotted in solid lines for reference

To evaluate the performance of the fabricated metasurfaces MS1 and MS2, we first characterize their PSF intensity profiles under two orthogonal circularly polarized incident states at a free-space wavelength of ${\lambda }_{0}=671$ nm. The measured PSF intensity profiles, along with their theoretical predictions, for the 0^th^-, 1^st^-, 2^nd^-, and 3^rd^-order differentiations are displayed in Fig. 3a–d. Except slight discrepancies, the measured PSFs exhibit good correspondence with theoretical predictions across all differentiation orders. Under LCP illumination, the PSF of MS1 exhibits a circularly symmetric solid-disk-shaped intensity distribution (Fig. 3a, left panel), with the full width at half-maximum (FWHM) value of its cross-sectional cut measuring 1.62 μm, closely matching the theoretical value of 1.67 μm (Fig. 3e, left panel). Under RCP illumination, the PSF instead displays a donut-shaped intensity distribution (Fig. 3a, right panel), where the peak-to-peak (PTP) distance in its cross-sectional cut is 1.92 μm, in excellent agreement with the theoretical value of 1.91 μm (Fig. 3e, right panel). For MS2, both PSF intensity profiles exhibit a hollow, ring-like spatial distribution (Fig. 3b), indicating suppression of low spatial-frequency components by the dark central region and preservation of high spatial-frequency contents by the bright outer ring region. The PTP distances in the cross-sectional cuts are respectively 3.44 μm (2^nd^-order differentiation) and 5.09 μm (3^rd^-order differentiation), in good correspondence to the simulated values of 3.33 μm and 5.05 μm (Fig. 3f). The experimentally measured focal lengths for the four cases, 4.809 ± 0.003 mm (MS1 under LCP illumination), 4.808 ± 0.004 mm (MS1 under RCP illumination), 4.810 ± 0.002 mm (MS2 under LCP illumination), and 4.812 ± 0.002 mm (MS2 under RCP illumination), nominally match the design value of 4.770 mm. The cited uncertainties represent three standard deviations of the measured data. The detailed procedure for the focal length characterization is elaborated in Materials and Methods.

**Fig. 4:**
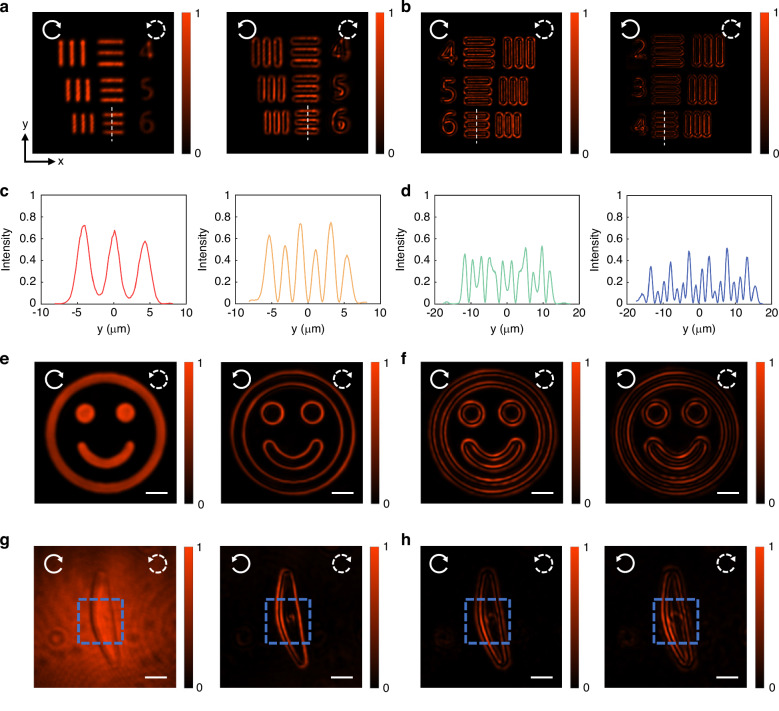
Imaging characterization of the fabricated spin-multiplexed metasurface differentiators at illumination wavelength of 671 nm. **a** The 0^th^-order (left panel) and 1^st^-order (right panel) differentiation image of the elements 4-6 of line pair group #7 of the 1951 USAF resolution test chart. **b** The 2^nd^-order (left panel) and 3^rd^-order (right panel) differentiation image of the elements 4-6 and elements 2-4 of line pair group #6 of the 1951 USAF resolution test chart. **c** The cross-sectional plot of the element 6 along the vertical direction in Fig. 4a. **d** The cross-sectional plot of the element 6 and element 4 along the vertical direction in Fig. 4b. **e–f** Imaging results of an amplitude-type object (custom-made binary metallic pattern) by device MS1 (**e**) and MS2 (**f**). Scale bars for Fig. 4e and f: 10 μm. Imaging results of a phase-type object (transparent diatom cell) by device MS1 (**g**) and MS2 (**h**). Scale bars for Fig. 4g and h: 10 μm. The solid and dash arrows in the top part of each figure represent the polarization states of the incident light and transmitted light, respectively. The clockwise arrow denotes LCP, while the counterclockwise arrow denotes RCP

To quantify the imaging resolution of the metasurface differentiator, we employ the 1951 United States Air Force (1951 USAF) resolution test chart as the target object. The measured 0^th^-order (MS1 under LCP illumination) and 1^st^-order (MS1 under RCP illumination) differential imaging results for elements 4 to 6 of line pair group #7, respectively with resolution (lp/mm) of 181.0 lp/mm, 203.0 lp/mm, and 228.0 lp/mm, are displayed in Fig. 4a. Element 6, corresponding to a line width of 2.19 μm, is clearly resolved (Fig. 4c). The measured 2^nd^-order (MS2 under LCP illumination) differential imaging results for elements 4 to 6 of line pair group #6 and 3^rd^-order (MS2 under RCP illumination) differential imaging results for elements 2 to 4 of line pair group #6 are displayed in Fig. 4b. Element 6 (corresponding to a line width of 4.39 μm) and element 4 (corresponding to a line width of 5.52 μm), are respectively resolved under the two modes, with two and three peaks generated for each edge of the line pair pattern (Fig. 4d). The experimentally measured imaging resolutions under different operational modes are largely consistent with the FWHM value or the PTP distance of the corresponding PSFs. It is worth noting that higher spatial resolutions can be readily achieved by designing devices with larger numerical aperture (NA) values. For instance, a metasurface differentiator with NA = 0.6 can achieve a spatial resolution of up to 456.0 lp/mm in its 0^th^- and 1^st^-order mode and 181.0 lp/mm in its 2^nd^- and 3^rd^-order mode. Such resolution respectively corresponds to element 6 of line pair group #8 and element 4 of line pair group #7 in the 1951 USAF resolution test chart. A detailed discussion is elaborated in Section S7, Supplementary Information. The operational efficiencies, defined as the ratio of the transmitted optical power to the total power illuminating the metalens, are measured to be 53.67 ± 1.06% for 0^th^-order imaging, 44.61 ± 1.54% for 1^st^-order differentiation, 41.27 ± 0.16% for 2^nd^-order differentiation, and 40.52 ± 0.60% for 3^rd^-order differentiation. The cited uncertainties represent three standard deviations of the measured data.

Next, we conduct imaging experiments on both amplitude-type object (custom-made binary metallic pattern on a coverslip) and phase-type object (transparent diatom cell). As shown in Fig. 4e–f, the amplitude-type object can be clearly imaged under multi-order differentiations, where the corresponding edge morphologies are effectively extracted. For the transparent phase-type objects, where light passes through with minimal intensity variation, the 0^th^-order differentiation (similar to conventional bright-field imaging) preserves the original scene information. This provides a complete view of the diatom cell, serving as a benchmark for comparison with higher-order differentiation results. In contrast, the 1^st^-order differentiation emphasizes high-frequency information, highlighting regions with rapid change in the light field. This mode effectively distinguishes the object from its background and delineates all the edges and boundaries within the full field of view. As indicated by the blue dashed square in Fig. 4g, the diatom nucleus, which is not clearly visible under the 0^th^-order differentiation due to its low intensity contrast, becomes clearly resolved in the 1^st^-order mode, providing a new perspective for cell identification. The higher-order differentiation results are displayed in Fig. 4h. In contrast to the 2^nd^-order differentiated image, the 3^rd^-order image reveals finer details in the intracellular region, particularly in the cytoplasm. However, the edges of the nucleus appear less distinct in the 3^rd^-order image. This contrast indicates that while higher-order differentiation enhances the visibility of subtle intracellular features, it may reduce the sharpness of larger structural boundaries like the nucleus. Hence, capturing all the subtle features during biological imaging may require a combination of different differentiation modalities, a capability conveniently offered by the metasurface-optics based platform.

### Comparison with conventional digital image processing

**Fig. 5:**
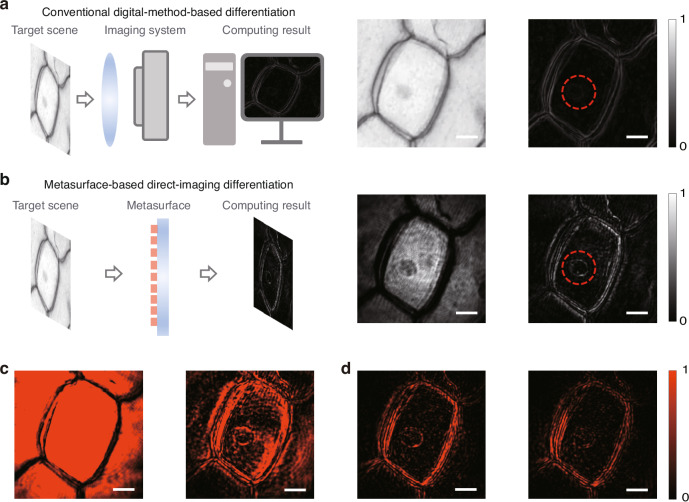
Comparison with conventional digital image processing and imaging performance under intense illumination conditions. **a** Left panel: Schematic representation of the conventional digital-method-based differentiation process, where the target scene is first captured by an imaging system, and the resulting image is subsequently processed by digital methods. Middle panel: The bright-field image of an onion epidermal cell captured by a commercial microscope. Right panel: The 1^st^-order differentiation image processed by a digital computer using a Sobel differential operator. **b** Left panel: Schematic representation of the metasurface-based simultaneous high-resolution imaging and optical differentiation process, where the metasurface performs both imaging and computational operations simultaneously and directly over the target scene. Middle panel: Image of the same onion epidermal cell captured by MS1 under its 0^th^-order differentiation mode. Right panel: Image captured by MS1 under its 1^st^-order differentiation mode. The red dashed circles in a and b denote the nucleus region of the onion epidermal cell. Scale bars for Fig. 5a and b: 20 μm. **c** Images captured by MS1 respectively under its 0^th^- (left panel) and 1^st^-order (right panel) differentiation modes under intense light illumination. **d** Images captured by MS2 respectively in its 2^nd^- (left panel) and 3^rd^-order (right panel) differentiation under intense light illumination. Scale bars for Fig. 5c and d: 20 μm

In conventional digital image processing, a target scene is first captured by an imaging system, after which computational operations are performed on the digitized image using electronic processors (Fig. 5a, left panel). The captured image typically encodes the scene’s intensity distribution in a matrix form, upon which various operators or solvers are applied. This approach, however, faces inherent limitations in terms of processing speed, throughput, and energy efficiency. For example, when the image size increases by a factor of N, the processing time generally scales as N²^55^. In contrast, our metasurface differentiator performs imaging and computational operations simultaneously on the optical field of the target scene, without additional processing overhead (Fig. 5b, left panel). This all-optical architecture bypasses the fundamental bottlenecks of digital methods. Furthermore, its processing speed remains essentially invariant—independent of image size or complexity—as long as the scene lies within the metasurface’s field of view.

We perform imaging experiments on the same target object using both a high-end commercial microscope and the fabricated metasurface (MS1). The bright-field image of an onion epidermal cell captured by the commercial microscope is displayed in the middle panel of Fig. 5a. By processing this image on a personal computer (Intel Core i7 10700 F processor) using Sobel differential operator, the 1^st^-order differentiation image is obtained (Fig. 5a, right panel). In comparison, the middle panel of Fig. 5b shows the image of the same onion cell captured by MS1 under its 0^th^-order differentiation mode, which closely matches the one taken by the commercial microscope. The right panel of Fig. 5b shows the image captured by MS1 under its 1st-order differentiation mode, which reveals richer details, particularly in the nucleus region, compared to the image processed by the computer. In the conventional approach, the camera-captured image only contains intensity information. Due to the relatively low intensity contrast between the onion nucleus and its surrounding cytoplasm, digital differentiation fails to resolve the nucleus’s edges, leading to information loss. In contrast, the metasurface-enabled direct-imaging approach performs in situ differentiation on the light field. Despite the low intensity contrast, the nucleus exhibits phase differences relative to its surroundings due to variations in their refractive indices and thicknesses. These phase differences are effectively captured and processed by the metasurface, allowing for an accurate differentiation. Our proposed direct-imaging-based computing method not only overcomes the limitations of conventional digital image processing methods by simplifying the signal transmission pathway but also minimizes information loss by performing differentiation directly over the entire light field, preserving both the amplitude and phase information. This enables the instantaneous acquisition and processing of multi-dimensional light field data, rather than being limited to intensity information alone.

Finally, we demonstrate the unique advantages of parallel processing of multidimensional light field information for imaging and sensing applications under intense illumination conditions. When the illumination intensity is sufficient to saturate an image detector during conventional bright-field imaging, we utilize MS1 and MS2 to image the same scene. Figures 5c and d show the images captured by these metasurfaces under two orthogonal circular polarization states. As expected, the image captured by MS1 under its 0^th^-order differentiation mode resembles typical bright-field imaging and is highly saturated (Fig. 5c, left panel), making it difficult to observe any intracellular structures of the onion cell. This underscores a key limitation of conventional imaging architectures: when the intensity distribution is first captured by an imaging system, saturation at this initial stage prevents subsequent image processing from recovering or extracting any meaningful information.

Our proposed architecture addresses this limitation by simultaneously capturing and processing both the amplitude and phase information of the target scene. As shown in Fig. 5c–d, images of different differentiation orders are generated by performing operations on the light field itself, rather than just on intensity. This allows regions with variations in intensity, phase, or both to be highlighted, enabling features like the cell nucleus and other intracellular structures to remain discernable even under saturated illumination. We also note that, as the differentiation order increases, the overall brightness level of the output image decreases. This is because a higher-order differentiator exhibits a PSF with a larger hollow central region, leading to a greater suppression of low spatial-frequency components in the imaging scene, thus lowering the brightness of the output image.

### Real-time imaging of live cells

**Fig. 6:**
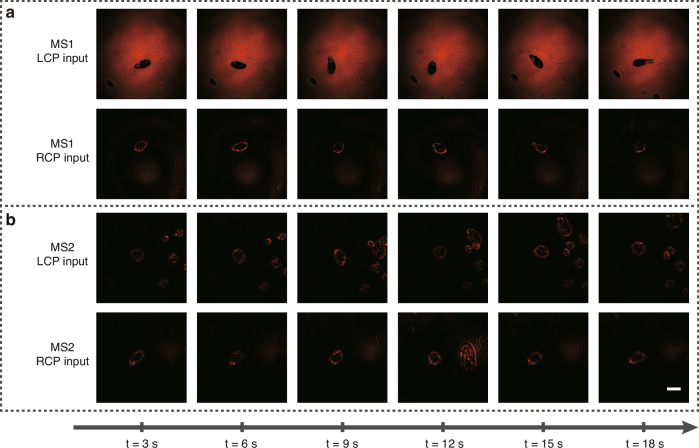
Real-time imaging of live Euglena cells. **a** Selected raw frames of the recorded video (Supplementary Movie 1) of live Euglena cells, captured by MS1 under its two differentiation modes. **b** Selected raw frames of the recorded video (Supplementary Movie 2) of live Euglena cells, captured by MS2 under its two differentiation modes. Scale bars for all figures: 20 μm

The proposed all-optical differentiator enables real-time computing and analysis of target objects, providing immediate and continuous examination of dynamic imaging scenes. Such capability presents distinct advantages for applications such as live cell observation and high-speed signal processing. To demonstrate this, we employ metasurfaces MS1 and MS2 to record videos of live Euglena cells. Figure 6 displays selected raw frames of the videos captured by each device under LCP and RCP illumination. The complete videos are provided in Supplementary Movies 1 and 2. The frame rate of the captured videos are set to be 30 fps. These results reveal differences in cell behavior as captured by each metasurface, highlighting the effectiveness of our approach. The 0^th^-order differentiation emphasizes the overall structure of the Euglena cells, allowing for clear visualization of their movement. In contrast, the 1^st^-order differentiation enhances cell edges, making it easier to detect rapid changes in their contours as the cells move or change shapes. Higher-order differentiations, such as 2^nd^- and 3^rd^-order, reveal greater details within the cells’ internal structures. These distinctions demonstrate that each differentiation mode provides a unique perspective, collectively offering a multifaceted view of the cells’ intricate behaviors.

### Broadband response of the metasurface differentiators

**Fig. 7:**
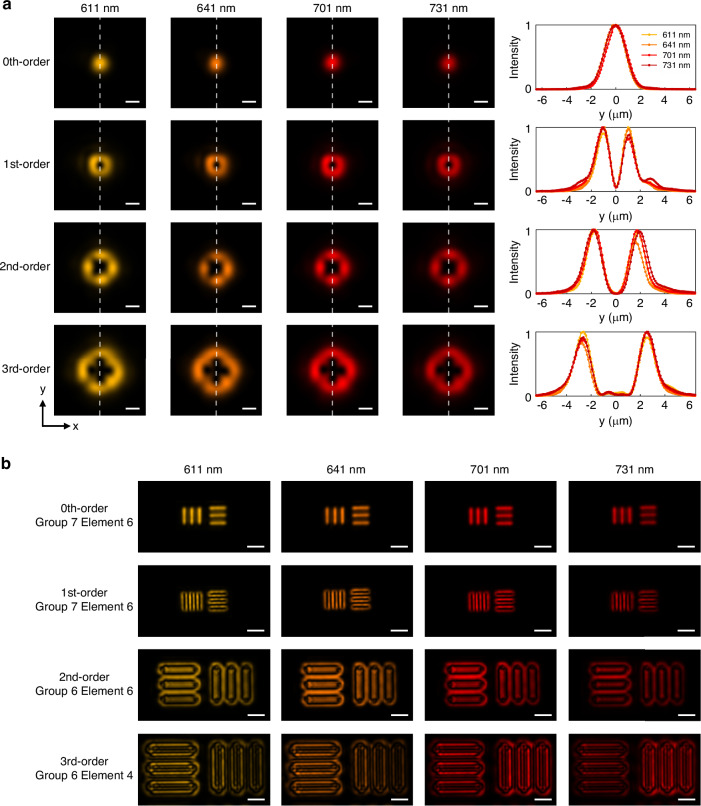
Broadband response of the fabricated spin-multiplexed metasurface differentiators. **a** Measured PSF intensity profiles (normalized) and the associated cross-sectional plots for the 0^th^-, 1^st^-, 2^nd^-, and 3^rd^- differentiations at illumination wavelengths of 611 nm, 641 nm, 701 nm and 731 nm. Scale bar: 2 μm. **b** Imaging results of the 1951 USAF resolution test chart at the tested illumination wavelengths. Scale bar: 10 μm

Finally, we characterize the performance of the fabricated devices across a broad wavelength range. The measured PSF intensity profiles for the 0^th^-, 1^st^-, 2^nd^-, and 3^rd^-order differentiations at illumination wavelengths of 611 nm, 641 nm, 701 nm, and 731 nm are shown in Fig. 7a. The associated focal lengths are listed in Table S1, Supplementary Information. The FWHM value or peak-to-peak (PTP) distances of the PSFs under different operational modes exhibit minimal variation across the tested wavelengths, indicating that the imaging resolution remains nearly constant. We further employ the 1951 USAF resolution test chart as the target object to quantify the imaging resolution. The devices resolve the same line pairs as observed at 671 nm. The measured 0^th^-, 1^st^-, 2^nd^-, and 3^rd^-order differential imaging results, respectively for elements 6 of group #7 (228.0 lp/mm), elements 6 of group #7 (228.0 lp/mm), elements 6 of group #6 (114.0 lp/mm), and elements 4 of group #6 (90.5 lp/mm), are displayed in Fig.7b. A detailed discussion on why the device maintains its imaging performance across different wavelengths is provided in Section S8, Supplementary Information.

## Discussion

The work presented here details a new class of all-optical differentiators based on single-layer function-multiplexed metasurfaces, enabling simultaneous integration of high-resolution imaging and multi-order differentiation. By independently modulating the phase and amplitude of two orthogonal circularly polarized light states, the metasurface’s complex-valued PSFs are precisely engineered to achieve various differentiation orders via simple polarization switching. Unlike traditional approaches such as the 4-*f* system or Green’s function methods, which are often constrained by system complexity, limited spatial resolution, or restricted functionality, our design eliminates the need for additional lenses or imaging modules while enabling multi-order differentiation with a single metasurface. Compared to recent vortex-phase-based methods, our PSF-engineering approach provides differentiation capability that is free from limitations on order and numerical aperture (i.e., spatial resolution), and at the same time, offers multiplexing flexibility. Experimental results of the implemented devices closely match theoretical predictions. Imaging under intense illumination conditions and real-time live cell observations further showcase the differentiator’s unique strengths in real-time light field capturing and processing capabilities across diverse application scenarios. Building on this general PSF-engineering strategy, we anticipate that other advanced all-optical computing functionalities—such as denoising and edge-enhancing operations—can be readily implemented (Section S9, Supplementary Information). Furthermore, the proposed design methodology is universal and can be adapted for other wavelength regions by selecting appropriate dielectric materials (e.g., hafnium oxide (HfO_2_)^56^ and tantalum pentoxide (Ta_2_O_5_)^57^ for UV devices, and titanium dioxide (TiO_2_)^58^, silicon nitride (SiN)^59^ and gallium nitride (GaN)^60^ for visible devices). This work offers a transformative approach to compact, high-performance all-optical computing devices, poised to support an array of applications in biological imaging, clinical diagnostics, and advanced signal processing.

## Materials and methods

### Device fabrication

The device fabrication begins with the deposition of a 530-nm-thick amorphous silicon (a-Si) film on a 500-μm-thick, double-side-polished fused silica substrate using plasma-enhanced chemical vapor deposition (PECVD). Next, a 300-nm-thick electron-beam (e-beam) resist layer is spin-coated onto the substrate, followed by deposition of a 20-nm-thick thermally-evaporated Al layer for anti-charging during the subsequent e-beam lithography process. The metasurface pattern is then defined in the e-beam resist layer through e-beam exposure, Al anti-charging layer removal, and resist development. Subsequently, a 20-nm-thick Cr layer is deposited via e-beam evaporation and patterned through a lift-off process. The patterned Cr layer is utilized as an etching mask to transfer the pattern into the a-Si layer using inductively coupled plasma reactive ion etching (ICP-RIE). Finally, the fabrication concludes with the removal of the remaining Cr etching mask and a thorough cleaning of the sample.

### Device characterization

To characterize the PSF of the metasurface, we use a supercontinuum laser, whose output is modulated and filtered by an acousto-optic tunable filter (AOTF) system, as the light source. A Köhler illumination setup is utilized to provide uniform illumination. Polarizers and quarter-wave plates, which operate at the target operational wavelength, are manually rotated to modulate the polarization state of the incident light. The intensity distribution at the metasurface’s focal plane is captured with a custom-built microscopy imaging system consisting of an NA = 0.7 objective, a tube lens, and an sCMOS camera. The system magnification is calibrated by translating the focal spot within the objective’s field of view using a high-precision stage. The physical size of the PSF projected by the metasurface is determined from the calibrated magnification and the pixel size of the sCMOS camera. To characterize the focal length of the metasurface, the device is initially placed on the focal plane of the microscopy imaging system’s objective lens. The metasurface is then translated perpendicularly away from the objective until its focal spot is sharply imaged by the microscopy system. The measured translation distance corresponds to the focal length of the metasurface. For the metasurface-differentiator-based imaging experiments, we use the same illumination setup. The target object is positioned at the metasurface’s focal plane, and its images are projected onto the sCMOS camera through a tube lens.

## Supplementary information

Supplementary Material for Spin-multiplexed Point Spread Function Engineering via Dielectric Metasurface for Simultaneous Optical Differentiation and High-resolution Imaging

Supplementary Material Movie S1

Supplementary Material Movie S2

## Data Availability

The data that support the findings of this study are available from the corresponding author upon reasonable request.
