# Supplementary material for: Spin-multiplexed point spread function engineering via dielectric metasurface for simultaneous optical differentiation and high-resolution imaging
Source: Light Sci Appl. 2026 Jul 15;15:318. doi: 10.1038/s41377-026-02229-1 (PMC13372813; doi:10.1038/s41377-026-02229-1)
Supplement: Supplementary file 1 — Supplementary Material for Spin-multiplexed Point Spread Function Engineering via Dielectric Metasurface for Simultaneous Optical Differentiation and High-resolution Imaging [file 41377_2026_2229_MOESM1_ESM.pdf]

Supplementary Information for

Spin-multiplexed Point Spread Function Engineering via Dielectric Metasurface for Simultaneous Optical Differentiation and High-resolution Imaging

Niu Liu[1†], Zhelin Lin[1†], Zhenyu Xing[1], Yuhui Hu[1], Yuxuan Liao[1], Amit Agrawal[2, 3], Xinliang Zhang[1*], and Cheng Zhang[1*]

[1]School of Optical and Electronic Information & Wuhan National Laboratory for Optoelectronics, Huazhong University of Science and Technology, Wuhan 430074, China

[2]Department of Engineering, University of Cambridge, Cambridge CB3 0FA, U.K.

[3]Kyung Hee University, 26 Kyungheedae-ro, Dongdaemun-gu, Seoul 02447, Korea

[†] Equal contributors

*Corresponding author Email: xlzhang@mail.hust.edu.cn; cheng.zhang@hust.edu.cn

Supplementary Text

Section S1. PSF engineering of the metasurface

To determine the point spread function (PSF) of the metasurface which performs simultaneous optical differentiation and high-resolution imaging, we consider the case where a point source S is located at the coordinate origin $(0, 0)$ of the object plane as the target object (Fig. S1). This point source S results in a spherical wave illuminating the metasurface plane.

$$\tilde{E}(x, y) = A\exp\left[ik_0\left(\frac{x^2 + y^2}{2l}\right)\right] \tag{S1}$$

where (x, y) is the two-dimensional (2D) coordinate at the metasurface plane; $k_0 = \frac{2\pi}{\lambda_0}$ is the free-space wave vector; λ_0 is the free-space operational wavelength of the metasurface; l is the distance from the metasurface to the object; and A is a constant. The metasurface implemented in this work functions as both a computing and an imaging element, with its computational capability enhanced by the spin-multiplexed response. Therefore, the overall light-field modulation (i.e., the transmission function) implemented via the metasurface is obtained by combining the pupil function and the focusing function:

$$\begin{aligned} T_{1,2}(x, y) = P_{1,2}(x, y) \cdot H(x, y) = E_{1,2}^{P}(x, y)exp\left[i\varphi_{1,2}^{P}(x, y)\right] \cdot exp[i\varphi_{lens}(x, y)] \\ = E_{1,2}(x, y)\,exp\left[i\varphi_{1,2}(x, y)\right] \end{aligned} \tag{S2}$$

where $T_{1,2}(x, y)$ is the target transmission function of the metasurface; $P_{1,2}(x, y)$ is the pupil function at the metasurface plane; $H(x, y)$ is the focusing function; $\varphi_{lens} = -\frac{2\pi}{\lambda_0}\left(\sqrt{{f_0}^2 + x^2 + y^2} - f_0\right)$ is the hyperbolic focusing phase; f_0 is the focal length of the metasurface; $E_{1,2}(x, y)$ and $\varphi_{1,2}(x, y)$ are the target amplitude and phase distribution implemented by the metasurface, respectively. The subscripts 1 and 2 correspond to the target

differentiation operation 1 and 2 under LCP and RCP illumination, respectively. Considering paraxial approximation, the transmission function satisfies

$$T_{1,2}(x,y) = P_{1,2}(x,y) \cdot \exp\left[-ik_0\left(\frac{x^2+y^2}{2f_0}\right)\right] \tag{S3}$$

Then the optical field distribution $\tilde{E}'_{1,2}(x,y)$ upon transmitting through the metasurface satisfies

$$\begin{aligned}\tilde{E}'_{1,2}(x,y) = \tilde{E}(x,y)\cdot T_{1,2}(x,y) &= A\exp\left[ik_0\left(\frac{x^2+y^2}{2l}\right)\right]\cdot P_{1,2}(x,y)\cdot \exp\left[-ik_0\left(\frac{x^2+y^2}{2f}\right)\right] \\ &= AP_{1,2}(x,y)\exp\left[-ik_0\left(\frac{x^2+y^2}{2l'}\right)\right] \end{aligned}\tag{S4}$$

where l' is the distance from the metasurface to the image and satisfies $\frac{1}{l'} = \frac{1}{f} - \frac{1}{l}$. The PSF distribution at the image plane of the metasurface satisfies

$$\begin{aligned} PSF_{1,2}(x_1,y_1) &= \iint \tilde{E}'_{1,2}(x,y)\cdot \exp\left[ik_0\frac{(x_1-x)^2+(y_1-y)^2}{2l'}\right]dxdy \\ &= \iint AP_{1,2}(x,y)\exp[-ik_0(\frac{x^2+y^2}{2l'})]\cdot \exp[ik_0\frac{(x_1-x)^2+(y_1-y)^2}{2l'}]dxdy \\ &= A\exp\left[ik_0\left(\frac{x_1^2+y_1^2}{2l'}\right)\right]\cdot \iint P_{1,2}(x,y)\exp\left[-ik_0\left(\frac{xx_1+yy_1}{l'}\right)\right]dxdy \\ &= A\exp\left[ik_0\left(\frac{x_1^2+y_1^2}{2l'}\right)\right]\cdot F\{P_{1,2}(x,y)\}|_{u=\frac{x_1}{\lambda_0 l'},v=\frac{y_1}{\lambda_0 l'}} \end{aligned}\tag{S5}$$

where (x_1,y_1) is the 2D coordinate at the image plane; (u,v) is the spatial frequency of the metasurface-modulated light field along the x- and y-axis, respectively; and F denotes the Fourier transform operation. Under the approximations of $x', y' \ll l'$ and $l' \approx f$, the point spread function satisfies

$$PSF_{1,2}(x_1,y_1) \propto F\{P_{1,2}(x,y)\}|_{u=\frac{x_1}{\lambda_0 f_0},v=\frac{y_1}{\lambda_0 f_0}} \tag{S6}$$

Through modification of the metasurface's pupil function, different complex-valued PSFs for differentiations of various orders can be engineered. The output light field distribution at the image

plane, $Img_{1,2}(x_1, y_1)$, is derived by convoluting the input light field at the object plane, $Obj_{1,2}(x_0, y_0)$, with the metasurface's PSF, $PSF_{1,2}(x_1, y_1)$.

$$\begin{aligned} Img_{1,2}(x_1, y_1) &= \iint_{-\infty}^{\infty} Obj_{1,2}(x_0, y_0)\, PSF_{1,2}(x_1 - x_0, y_1 - y_0) dx_0 dy_0 \\ &= Obj_{1,2}(x_0, y_0) \otimes PSF_{1,2}(x_1, y_1) \\ &\propto Obj_{1,2}(x_0, y_0) \otimes F\{P_{1,2}(x, y)\} \end{aligned} \tag{S7}$$

where (x_0, y_0) is the 2D coordinate at the object plane, $\otimes$ denotes the convolution operation. Furthermore, by applying the convolution process in Eq. S7 twice, the output satisfies

$$\begin{aligned} Img_{1,2}(x_1, y_1) &= \left[Obj_{1,2}(x_0, y_0) \otimes PSF_{1,2}(x_1, y_1)\right] \otimes PSF_{1,2}(x_1, y_1) \\ &\propto [Obj_{1,2}(x_0, y_0) \otimes F\{P_{1,2}(x, y)\}] \otimes F\{P_{1,2}(x, y)\} \\ &\propto Obj_{1,2}(x_0, y_0) \otimes \left[F\{P_{1,2}(x, y)\} \otimes F\{P_{1,2}(x, y)\}\right] \\ &\propto Obj_{1,2}(x_0, y_0) \otimes F\{P_{1,2}(x, y) \cdot P_{1,2}(x, y)\} \end{aligned} \tag{S8}$$

If a single convolution process implements a 1st-order differentiation operation, then the multiple convolutions yield higher-order differentiations. Eq. S8 indicates that each successive convolution is equivalent to a recursive multiplication of the pupil function associated with the 1st-order differentiation. Therefore, this recursive approach provides an effective method for implementing arbitrary order differentiations through repeated convolutions.

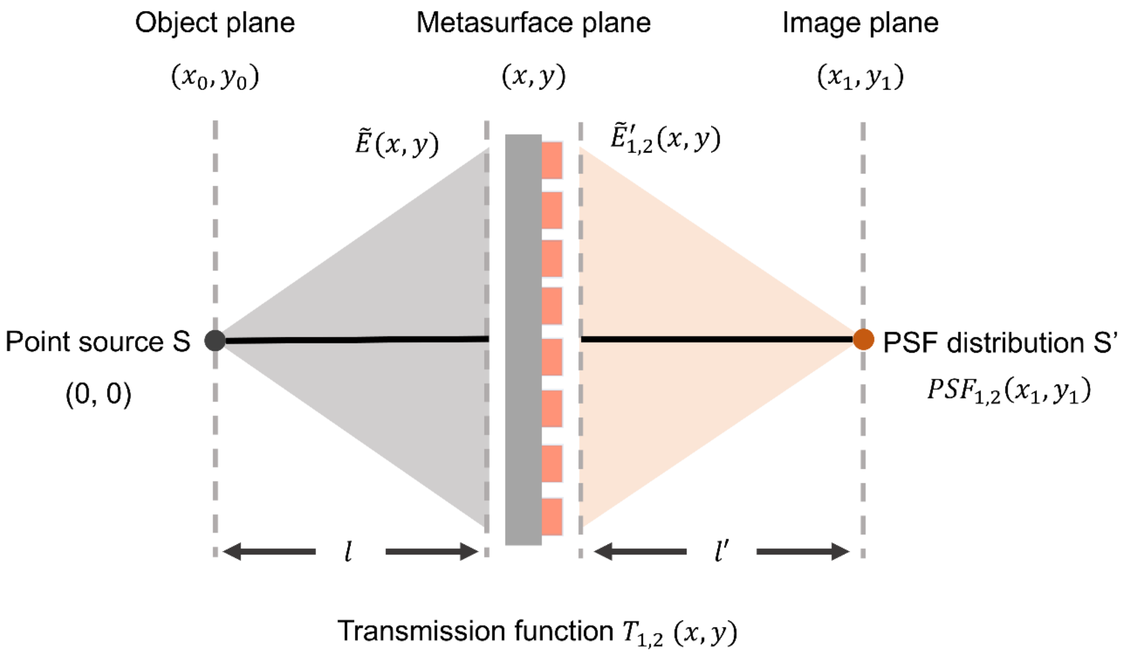

Fig. S1: Schematic representation of the PSF-engineered metasurface differentiator. A point source S is located at the coordinate origin $(0, 0)$ of the object plane, with a distance l from the metasurface plane. The metasurface results in a corresponding PSF distribution S' at the image plane, with a distance l' from the metasurface plane.

Section S2. Amplitude and phase distributions of the implemented metasurfaces

Figure S2 presents the target amplitude and phase distributions for the pupil functions, focusing functions, and transmission functions of the implemented metasurface differentiators, which are specifically engineered to realize simultaneous high-resolution imaging and optical differentiations of 0th-, 1st-, 2nd-, and 3rd-order.

For the 0th-order differentiation, the pupil function exhibits a constant phase distribution and Gaussian-like amplitude distribution. For higher-order differentiations, the pupil functions exhibit vortex-like phase distributions and hollow-core amplitude distributions. The focusing functions for all above cases are kept identical, ensuring that the computed imaging outputs are generated at the same image plane for the same field of view. The target transmission functions, which represent the desired complex light modulation at the metasurface plane, are the combination of the complex-valued pupil functions and the focusing functions. Once its transmission functions are determined, the metasurface can then be designed to provide simultaneous phase and amplitude modulations, enabling integration of high-resolution imaging and differentiations of multi-orders over the target object.

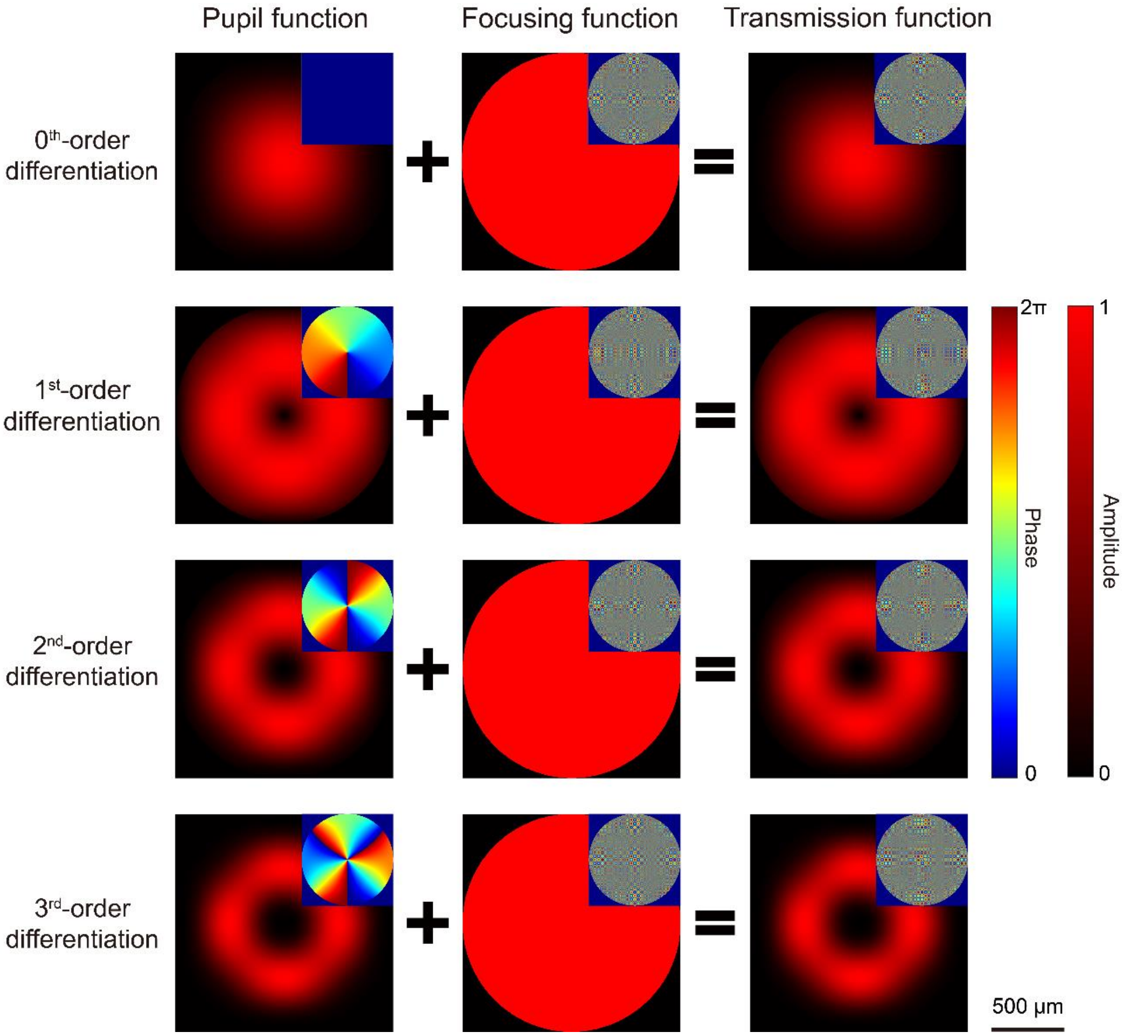

Fig. S2: Amplitude and phase (inset) distributions of the implemented metasurface differentiators to realize simultaneous high-resolution imaging and multi-order differentiations. The target transmission function implemented via the metasurface is the combination of the complex-valued pupil function and the focusing function.

Section S3. Meta-atom design

The light field modulation property of the metasurface can be represented by a Jones matrix $J(x, y)$

$$\begin{cases} E_1(x, y)\mathrm{e}^{i\cdot\varphi_1(x,y)}|RCP\rangle = J(x, y)|LCP\rangle \\ E_2(x, y)\mathrm{e}^{i\cdot\varphi_2(x,y)}|LCP\rangle = J(x, y)|RCP\rangle \end{cases} \tag{S9}$$

where $|LCP\rangle = \frac{\sqrt{2}}{2}[1 \quad i]^T$ and $|RCP\rangle = \frac{\sqrt{2}}{2}[1 \quad -i]^T$ are the Jones vectors representing LCP and RCP light, respectively. $E_1(x, y)\mathrm{e}^{i\cdot\varphi_1(x,y)}$ and $E_2(x, y)\mathrm{e}^{i\cdot\varphi_2(x,y)}$ represent the target complex amplitude modulations under LCP and RCP illumination, respectively. Based on Eq. S9, the Jones matrix $J(x, y)$ satisfies

$$J(x, y) = \begin{bmatrix} E_1(x, y)\mathrm{e}^{i\cdot\varphi_1(x,y)} + E_2(x, y)\mathrm{e}^{i\cdot\varphi_2(x,y)} & -i(E_1(x, y)\mathrm{e}^{i\cdot\varphi_1(x,y)} - E_2(x, y)\mathrm{e}^{i\cdot\varphi_2(x,y)}) \\ -i(E_1(x, y)\mathrm{e}^{i\cdot\varphi_1(x,y)} - E_2(x, y)\mathrm{e}^{i\cdot\varphi_2(x,y)}) & -E_1(x, y)\mathrm{e}^{i\cdot\varphi_1(x,y)} - E_2(x, y)\mathrm{e}^{i\cdot\varphi_2(x,y)} \end{bmatrix} \tag{S10}$$

The target complex amplitude modulation can be further decomposed as

$$\begin{cases} E_1(x, y)\mathrm{e}^{i\cdot\varphi_1(x,y)} = \frac{1}{2}(\mathrm{e}^{i\cdot\varphi_A^+(x,y)} + \mathrm{e}^{i\cdot\varphi_B^+(x,y)}) \\ E_2(x, y)\mathrm{e}^{i\cdot\varphi_2(x,y)} = \frac{1}{2}(\mathrm{e}^{i\cdot\varphi_A^-(x,y)} + \mathrm{e}^{i\cdot\varphi_B^-(x,y)}) \end{cases} \tag{S11}$$

Combining Eq. S10 and Eq. S11, the Jones matrix $J(x, y)$ can be decomposed into the sum of two matrices

$$J(x, y) = \frac{1}{2}\left(J_A(x, y) + J_B(x, y)\right) \tag{S12}$$

where $J_A(x, y)$ and $J_B(x, y)$ respectively represent the Jones matrices of the meta-atoms A and B

$$J_A(x, y) = \frac{1}{2}\begin{bmatrix} \mathrm{e}^{i\cdot\varphi_A^+(x,y)} + \mathrm{e}^{i\cdot\varphi_A^-(x,y)} & -i\left(\mathrm{e}^{i\cdot\varphi_A^+(x,y)} - \mathrm{e}^{i\cdot\varphi_A^-(x,y)}\right) \\ -i\left(\mathrm{e}^{i\cdot\varphi_A^+(x,y)} - \mathrm{e}^{i\cdot\varphi_A^-(x,y)}\right) & -\mathrm{e}^{i\cdot\varphi_A^+(x,y)} - \mathrm{e}^{i\cdot\varphi_A^-(x,y)} \end{bmatrix} \tag{S13}$$

$$J_B(x, y) = \frac{1}{2}\begin{bmatrix} \mathrm{e}^{i\cdot\varphi_B^+(x,y)} + \mathrm{e}^{i\cdot\varphi_B^-(x,y)} & -i\left(\mathrm{e}^{i\cdot\varphi_B^+(x,y)} - \mathrm{e}^{i\cdot\varphi_B^-(x,y)}\right) \\ -i\left(\mathrm{e}^{i\cdot\varphi_B^+(x,y)} - \mathrm{e}^{i\cdot\varphi_B^-(x,y)}\right) & -\mathrm{e}^{i\cdot\varphi_B^+(x,y)} - \mathrm{e}^{i\cdot\varphi_B^-(x,y)} \end{bmatrix} \tag{S14}$$

$J_A(x, y)$ and $J_B(x, y)$ satisfy the following conditions

$$\begin{cases} J_A(x,y) = J_A(x,y)^T \\ J_B(x,y) = J_B(x,y)^T \\ J_A(x,y)J_A(x,y)^H = J_A(x,y)^H J_A(x,y) = I \\ J_B(x,y)J_B(x,y)^H = J_B(x,y)^H J_B(x,y) = I \end{cases} \tag{S15}$$

where T denotes the transpose operation; H denotes the conjugate transpose operation; and I is the identity matrix. Equation S15 demonstrates that $J_A(x,y)$ and $J_B(x,y)$ are symmetric and unitary, which indicates that their eigenvalues and eigenvectors can be determined by solving the characteristic equations. For $J_A(x,y)$, the two eigenvalues are

$$\begin{cases} \varphi_A^x(x,y) = \frac{1}{2}\left(\varphi_A^+(x,y) + \varphi_A^-(x,y)\right) \\ \varphi_A^y(x,y) = \frac{1}{2}\left(\varphi_A^+(x,y) + \varphi_A^-(x,y)\right) + \pi \end{cases} \tag{S16}$$

And the associated eigenvectors are

$$\begin{cases} V_1 = [\cos\theta_A(x,y) \quad \sin\theta_A(x,y)]^T \\ V_2 = [\cos\theta_A(x,y) \quad -\sin\theta_A(x,y)]^T \end{cases} \tag{S17}$$

According to Eq. S16 and S17, $J_A(x,y)$ can be represented as

$$J_A(x,y) = \begin{bmatrix} \cos\theta_A(x,y) & -\sin\theta_A(x,y) \\ \sin\theta_A(x,y) & \cos\theta_A(x,y) \end{bmatrix} \begin{bmatrix} e^{i\cdot\varphi_A^x(x,y)} & 0 \\ 0 & e^{i\cdot\varphi_A^y(x,y)} \end{bmatrix} \begin{bmatrix} \cos\theta_A(x,y) & \sin\theta_A(x,y) \\ -\sin\theta_A(x,y) & \cos\theta_A(x,y) \end{bmatrix} \tag{S18}$$

with $\varphi_A^x(x,y)$, $\varphi_A^y(x,y)$, and $\theta_A(x,y)$ satisfying

$$\begin{cases} \varphi_A^x(x,y) = \frac{1}{2}\left(\varphi_A^+(x,y) + \varphi_A^-(x,y)\right) \\ \varphi_A^y(x,y) = \frac{1}{2}\left(\varphi_A^+(x,y) + \varphi_A^-(x,y)\right) + \pi \\ \theta_A(x,y) = \frac{1}{4}\left(\varphi_A^+(x,y) - \varphi_A^-(x,y)\right) \end{cases} \tag{S19}$$

Similarly, $J_B(x,y)$ can be represented as

$$J_B(x,y) = \begin{bmatrix} \cos\theta_B(x,y) & -\sin\theta_B(x,y) \\ \sin\theta_B(x,y) & \cos\theta_B(x,y) \end{bmatrix} \begin{bmatrix} e^{i\cdot\varphi_B^x(x,y)} & 0 \\ 0 & e^{i\cdot\varphi_B^y(x,y)} \end{bmatrix} \begin{bmatrix} \cos\theta_B(x,y) & \sin\theta_B(x,y) \\ -\sin\theta_B(x,y) & \cos\theta_B(x,y) \end{bmatrix} \tag{S20}$$

with $\varphi_B^x(x,y)$, $\varphi_B^y(x,y)$, and $\theta_B(x,y)$ satisfying

$$\begin{cases} \varphi_B^x(x,y) = \frac{1}{2}\left(\varphi_B^+(x,y) + \varphi_B^-(x,y)\right) \\ \varphi_B^y(x,y) = \frac{1}{2}\left(\varphi_B^+(x,y) + \varphi_B^-(x,y)\right) + \pi \\ \theta_B(x,y) = \frac{1}{4}\left(\varphi_B^+(x,y) - \varphi_B^-(x,y)\right) \end{cases} \quad (S21)$$

Here, $\varphi_A^+(x,y)$, $\varphi_B^+(x,y)$, $\varphi_A^-(x,y)$, and $\varphi_B^-(x,y)$ can be further derived from the target complex amplitude modulation based on Eq. S11.

$$\begin{cases} \varphi_A^+(x,y) = \cos^{-1}\left(E_1(x,y)\right) + \varphi_1(x,y) \\ \varphi_B^+(x,y) = \varphi_1(x,y) - \cos^{-1}\left(E_1(x,y)\right) \\ \varphi_A^-(x,y) = \cos^{-1}\left(E_2(x,y)\right) + \varphi_2(x,y) \\ \varphi_B^-(x,y) = \varphi_2(x,y) - \cos^{-1}\left(E_2(x,y)\right) \end{cases} \quad (S22)$$

Based on the above analysis, different twin meta-atoms A and B can be selected to form the super cell at a given location (x,y) at the metasurface plane with the required anisotropic phase modulation values $\varphi_{A,B}^x(x,y)$ and $\varphi_{A,B}^y(x,y)$, as well as rotation angles $\theta_A(x,y)$ and $\theta_B(x,y)$, to implement the target complex amplitude modulation $E_1(x,y)\mathrm{e}^{i\cdot\varphi_1(x,y)}$ and $E_2(x,y)\mathrm{e}^{i\cdot\varphi_2(x,y)}$ under LCP and RCP illumination, respectively.

Section S4. The intensity transmission coefficient and phase shift of the meta-atoms

The intensity transmission coefficient (T_x and T_y) and phase shift (φ_x and φ_y) for propagation of 671 nm wavelength light, linearly-polarized along the major axis of the rectangular a-Si nanopillars with different cross-sectional dimensions (W, L) are computed using finite-difference time-domain (FDTD) simulations with periodic boundary conditions, as shown in Fig. S3. During the parameter sweeping process, the ranges of variation for both W and L are set to be [60 nm, 270 nm], with a step size of 1 nm.

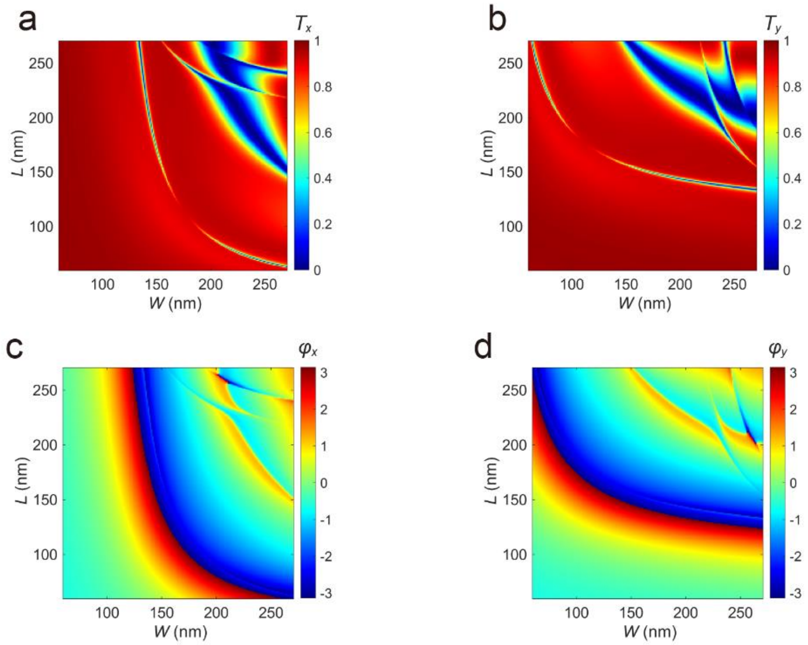

Fig. S3: Simulated intensity transmission coefficient and phase shift of the meta-atoms. (a-b) Simulated intensity transmission coefficients (T_x and T_y) versus a-Si meta-atom in-plane dimensions (W, L) at x-polarized and y-polarized free-space incident wavelength of $\lambda_0 = 671\ nm$. (c-d) Simulated phase shift modulation (φ_x and φ_y) versus a-Si meta-atom in-plane dimensions (W, L) at x-polarized and y-polarized free-space incident light of $\lambda_0 = 671\ nm$.

Section S5. The parameters of associated meta-atom structures used in the metasurface design

The implemented metasurface devices are composed of 40 nanopillars with distinct in-plane dimensions (W, L). The in-plane dimensions and FoM values of the selected meta-atoms are as follows.

Table S1. Parameters for the selected meta-atoms

No.	*W* (nm)	*L* (nm)	*FoM*	No.	*W* (nm)	*L* (nm)	*FoM*
1	60	245	-6.32	21	185	95	-6.15
2	75	205	-5.53	22	185	100	-6.87
3	80	200	-6.17	23	185	105	-8.22
4	90	190	-6.26	24	185	110	-4.91
5	95	185	-5.97	25	190	90	-5.96
6	100	185	-7.20	26	190	105	-3.99
7	105	185	-7.85	27	190	110	-6.70
8	105	190	-4.09	28	190	115	-3.83
9	110	185	-4.80	29	195	115	-5.51
10	110	190	-7.02	30	200	80	-5.88
11	115	190	-3.75	31	205	75	-5.65
12	115	195	-5.35	32	205	115	-5.39
13	115	205	-5.54	33	210	115	-4.26
14	115	215	-3.69	34	210	120	-3.19
15	115	220	-3.25	35	215	120	-3.55
16	120	225	-4.29	36	225	120	-4.33
17	120	235	-5.27	37	235	120	-5.33
18	120	245	-6.38	38	245	60	-6.24
19	120	260	-5.60	39	245	120	-6.40
20	120	215	-3.50	40	260	120	-5.39

Section S6. Fabrication robustness analysis

In this work, we implement two metasurface devices: one performing $0^{th}/1^{st}$-order differentiation and the other performing $2^{nd}/3^{rd}$-order differentiation. Each device has a diameter of D = 3.000 mm and numerical aperture of NA = 0.3, corresponding to a focal length of f_0 = 4.770 mm. To assess the robustness of the metasurface design, we numerically simulate devices with random cross-sectional width and length variations of ±5 nm, ±10 nm, and ±20 nm in their constituent meta-atoms.

For the 0th- and 1st-order multiplexed differentiators with random variation of ± 5 nm, ± 10 nm, and ± 20 nm, the simulated PSF intensity and phase profiles under LCP and RCP illuminations are displayed in the left panels of Figs. S4a to S4c, respectively. A line pair with resolution of 228.0 lp/mm, which equals to the resolution of element 6 of line pair group #7 in the 1951 USAF resolution test chart, can be clearly resolved under both the 0th-order and 1st-order differentiation modes (Right panels of Figs. S4a to S4c). The resulting device exhibits robust performance, maintaining high PSF fidelity and imaging quality.

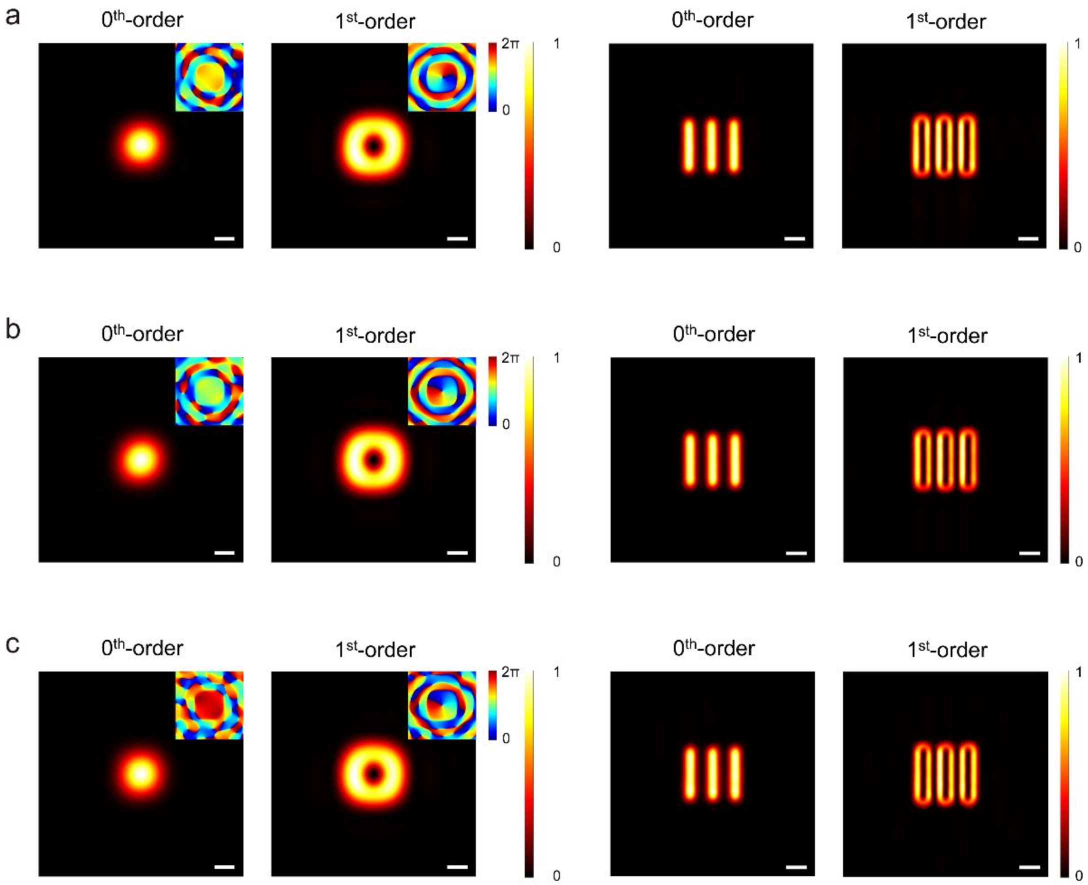

Fig. S4: Numerical evaluation of the performance of the 0th- and 1st-order multiplexed metasurface differentiators with random variation in the width and length of meta-atoms. (a) Left panel: Simulated complex-valued PSF distributions of the 0th-order (left panel) and 1st-order (right panel) multiplexed differentiator with random variations of ± 5 nm in the width and length of meta-atoms. Scale bar: 1 μm. Right panel: Corresponding simulated imaging results under the 0th-order (left panel) and 1st-order (right panel) differentiation modes. Scale bar: 2 μm. (b) Left panel: Simulated complex-valued PSF distributions of the 0th-order (left panel) and 1st-order (right panel) multiplexed differentiator with random variations of ± 10 nm in the width and length of meta-atoms. Scale bar: 1 μm. Right panel: Corresponding simulated imaging results under the 0th-order (left panel) and 1st-order (right panel) differentiation modes. Scale bar: 2 μm. (c) Left panel: Simulated complex-valued PSF distributions of the 0th-order (left panel) and 1st-order (right panel) multiplexed differentiator with random variations of ± 20 nm in the width and length of meta-atoms. Scale bar: 1 μm. Right panel: Corresponding simulated imaging results under the 0th-order (left panel) and 1st-order (right panel) differentiation modes. Scale bar: 2 μm.

For 2nd- and 3rd-order multiplexed differentiator with random variation of ± 5 nm, ± 10 nm, and ± 20 nm, the simulated PSF intensity and phase profiles under LCP and RCP illumination are

displayed in the left panels of Figs. S5a to S5c, respectively. Line pairs with resolution of 114.0 lp/mm and 90.5 lp/mm, can be clearly resolved under the 2nd-order and 3rd-order differentiation modes (right panels of Figs. S5a to S5c), respectively, demonstrating stable performance under tested fabrication variations.

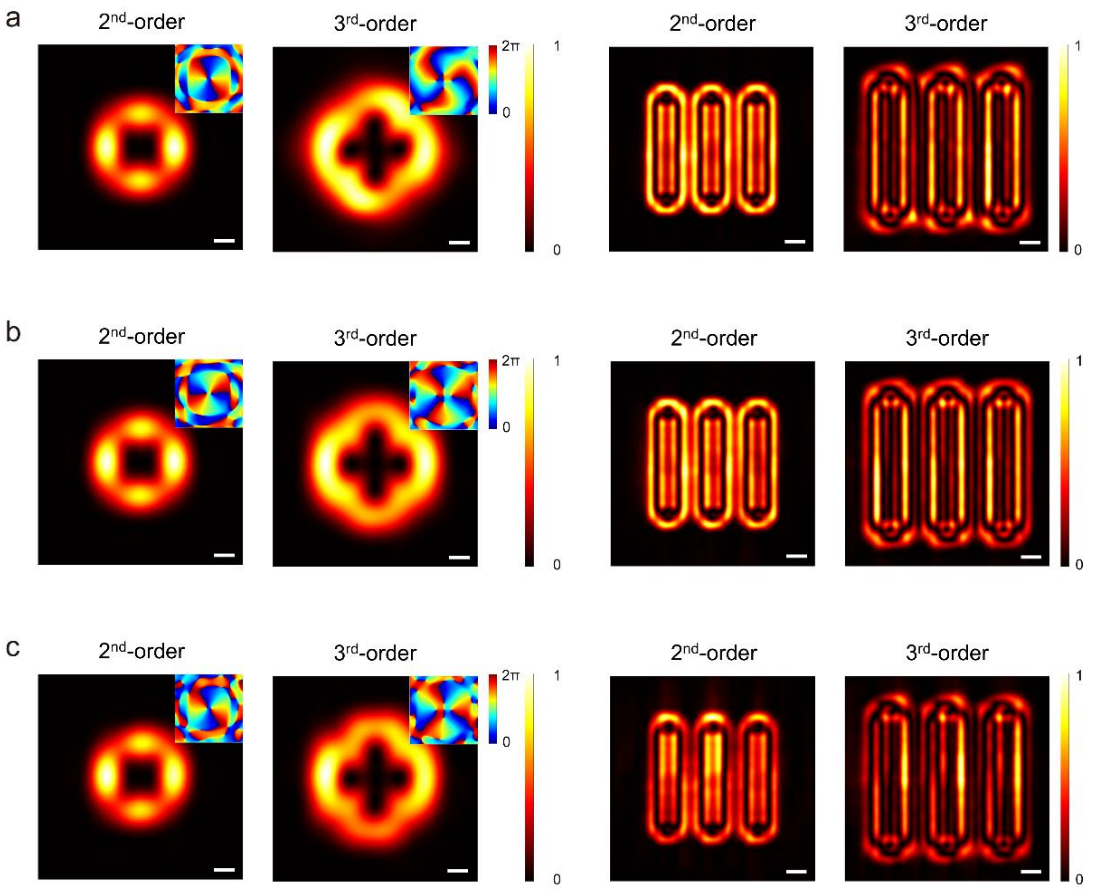

Fig. S5: Numerical evaluation of the performance of the 2nd- and 3rd-order multiplexed metasurface differentiators with random variation in the width and length of meta-atoms. (a) Left panel: Simulated complex-valued PSF distributions of the 2nd-order and 3rd-order multiplexed differentiator with random variations of ± 5 nm in the width and length of meta-atoms. Scale bar: 1 μm. Right panel: Corresponding simulated imaging results under the 2nd-order (left panel) and 3rd-order (right panel) differentiation modes. Scale bar: 2 μm. (b) Left panel: Simulated complex-valued PSF distributions of the 2nd-order and 3rd-order multiplexed differentiator with random variations of ± 10 nm in the width and length of meta-atoms. Scale bar: 1 μm. Right panel: Corresponding simulated imaging results under the 2nd-order (left panel) and 3rd-order (right panel) differentiation modes. Scale bar: 2 μm. (c) Left panel: Simulated complex-valued PSF distributions of the 2nd-order and 3rd-order multiplexed differentiator with random variations of ± 20 nm in the width and length of meta-atoms. Scale bar: 1 μm. Right panel: Corresponding simulated imaging results under the 2nd-order (left panel) and 3rd-order (right panel) differentiation modes. Scale bar: 2 μm.

Section S7. Metasurface differentiators of larger numerical aperture values

Unlike previous demonstrated approaches, our proposed design is based on PSF engineering and therefore, is not constrained by the obtained numerical aperture (NA) values. This allows for fine spatial resolution of the computed differentiation images. As a proof of concept, we design two different kinds of metasurface with the same NA = 0.6, each capable of performing $0^{th}/1^{st}$-order and $2^{nd}/3^{rd}$-order spin-multiplexed differentiations, respectively. Both devices have the same diameter (D = 1.000 mm), numerical aperture (NA = 0.6), and focal length (f_0 = 0.667 mm). The meta-atom library used to construct these metasurfaces is the same as the one for the devices in the main text.

We evaluate the performance of the constructed metasurfaces through numerical simulation. For the 0th- and 1st-order multiplexed differentiator, the simulated PSF intensity and phase profiles under LCP and RCP illuminations are displayed in Fig. S6a. A line pair with resolution of 456.0 lp/mm, which equals to the resolution of element 6 of line pair group #8 in the 1951 USAF resolution test chart, can be clearly resolved under both the 0th-order (Fig. S6b, left panel) and 1st-order differentiation (Fig. S6b, right panel) modes.

For 2nd- and 3rd-order multiplexed differentiator, the simulated PSF intensity and phase profiles under LCP and RCP illumination are displayed in Fig. S6c. A line pair with resolution of 181.0 lp/mm, which equal to the resolution of element 4 of line pair group #7 in the 1951 USAF resolution test chart, can be clearly resolved under both 2nd-order differentiation (Fig. S6d, left panel) and 3rd-order differentiation (Fig. S6d, right panel) modes.

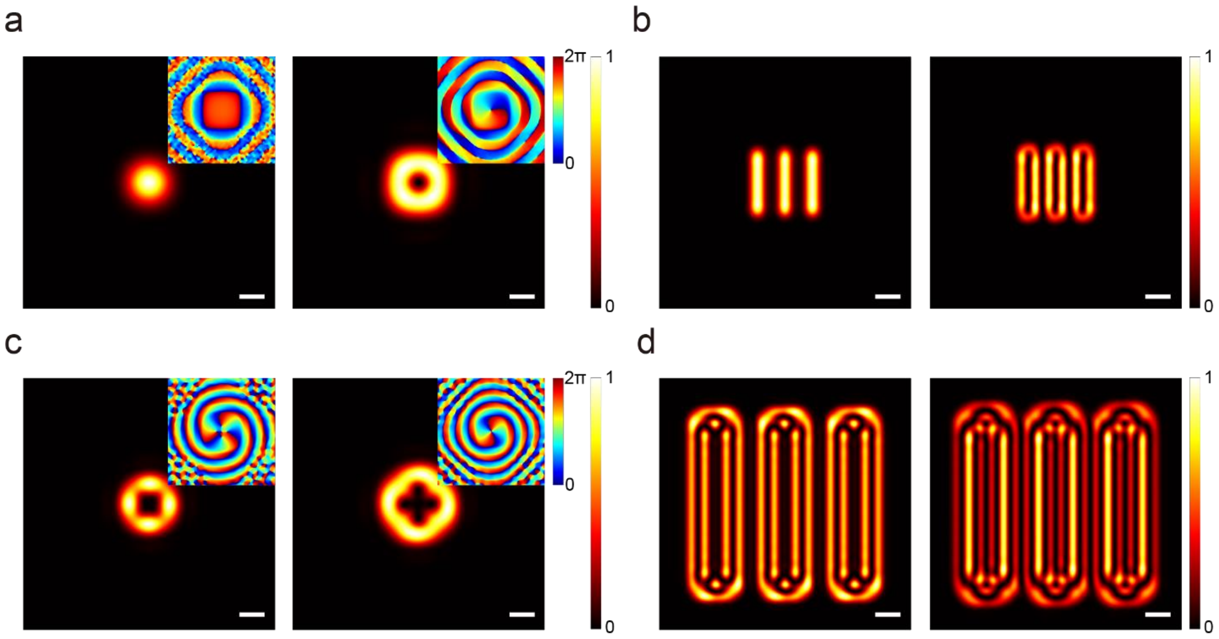

Fig. S6: Numerical evaluation of the performance of the multiplexed metasurface differentiators with numerical aperture of 0.6. (a) Simulated complex-valued PSF distributions of the 0th-order (left panel) and 1st-order (right panel) multiplexed differentiator. Scale bar: 1 μm. (b) Simulated imaging results under the 0th-order (left panel) and 1st-order (right panel) differentiation modes. A line pair with a spatial resolution of 456.0 lp/mm, which equals to the resolution of element 6 of the line pair group #8 in the 1951 USAF resolution test chart, can be clearly resolved. Scale bar: 2 μm. (e) Simulated complex-valued PSF distribution of the 2nd-order (left panel) and 3rd-order (right panel) multiplexed differentiator. Scale bar: 1 μm. (f) Simulated imaging results under the 2nd-order (left panel) and 3rd-order (right panel) differentiation modes. A line pair with a spatial resolution of 181.0 lp/mm, which equals to the resolution of element 4 of the line pair group #7 in the 1951 USAF resolution test chart, can be clearly resolved. Scale bar: 2 μm.

Section S8. Broadband response of the metasurface differentiators

The schematic representation of the metasurface differentiator under different illumination wavelengths is illustrated in Fig. S7. Due to the wavelength insensitivity of the Pancharatnam-Berry (PB) phase employed in the metasurface design, the amplitude and phase response remain consistent across a broad wavelength range and can be expressed as

$$T_{1,2}^{\lambda}(x,y) = T_{1,2}(x,y) = E^{P}(x,y)\exp[i\varphi^{P}(x,y)] \cdot \exp[i\varphi_{lens}(x,y)] \tag{S23}$$

where $T_{1,2}^{\lambda}(x,y)$ is the transmission function at the free-space operational wavelength λ. The devices, whose corresponding hyperbolic focusing function satisfying $\varphi_{lens} = -\frac{2\pi}{\lambda_0}\left(\sqrt{{f_0}^2 + x^2 + y^2} - f_0\right)$, are designed to operate at free-space wavelength λ_0 with a focal length of f_0. Under paraxial approximation, for a normally incident light of free-space wavelength λ, the focal length f_λ of the device can be expressed as [1]

$$f_\lambda = \frac{\lambda_0}{\lambda} f_0 \tag{S24}$$

We experimentally characterize the focal length of MS1 and MS2 at illumination wavelengths of 611 nm, 641 nm, 701 nm, and 731 nm, which are in good correspondence to the simulated values (Table S2). The cited uncertainties represent three standard deviations of the measured data.

Table S2. Measured focal lengths of the fabricated devices at the tested illumination wavelengths

λ	Target Value	0th-order	1st-order	2nd-order	3rd-order
611 nm	5.238 mm	5.253 ± 0.002 mm	5.255 ± 0.003 mm	5.256 ± 0.004 mm	5.255 ± 0.003 mm
641 nm	4.993 mm	5.000 ± 0.003 mm	5.003 ± 0.003 mm	5.003 ± 0.003 mm	5.001 ± 0.002 mm
701 nm	4.566 mm	4.563 ± 0.004 mm	4.561 ± 0.003 mm	4.560 ± 0.003 mm	4.561 ± 0.003 mm
731 nm	4.378 mm	4.367 ± 0.003 mm	4.363 ± 0.001 mm	4.368 ± 0.003 mm	4.369 ± 0.001 mm

Then the hyperbolic focusing phase can be rewritten as

$$\varphi_{lens} = -\frac{2\pi}{\lambda_0}\left(\sqrt{f_0^2 + x^2 + y^2} - f_0\right)$$

$$= -\frac{2\pi}{\lambda_0}\left(\sqrt{\frac{\lambda^2}{\lambda_0^2}{f_\lambda}^2 + x^2 + y^2} - \frac{\lambda}{\lambda_0}f_\lambda\right)$$

$$= -\frac{2\pi\lambda}{\lambda_0^2}\left(\sqrt{{f_\lambda}^2 + \frac{\lambda_0^2}{\lambda^2}(x^2 + y^2)} - f_\lambda\right)$$

$$\cong -\frac{2\pi\lambda}{\lambda_0^2}\left(\frac{\lambda_0^2(x^2 + y^2)}{2f_\lambda\lambda^2}\right)$$

$$= -\frac{2\pi}{\lambda}\frac{(x^2 + y^2)}{2f_\lambda} \qquad (S25)$$

Similar to Eq. S4, the optical field distribution $\widetilde{E'}^{\lambda}_{1,2}(x, y)$ upon transmitting through the metasurface satisfies

$$\widetilde{E'}^{\lambda}_{1,2}(x, y) = \tilde{E}(x, y) \cdot T^{\lambda}_{1,2}(x, y) = A\exp\left[i\frac{2\pi}{\lambda}\left(\frac{x^2 + y^2}{2l}\right)\right] \cdot P_{1,2}(x, y) \cdot \exp\left[-i\frac{2\pi}{\lambda}\frac{(x^2 + y^2)}{2f_\lambda}\right]$$

$$= AP_{1,2}(x, y)\exp\left[-i\frac{2\pi}{\lambda}\left(\frac{x^2 + y^2}{2{l_\lambda}'}\right)\right] \qquad (S26)$$

where ${l_\lambda}'$ is the distance from the metasurface to the image and satisfies $\frac{1}{{l_\lambda}'} = \frac{1}{f_\lambda} - \frac{1}{l}$. The PSF distribution at the image plane of the metasurface satisfies

$$PSF^{\lambda}_{1,2}(x_1, y_1) = \iint \widetilde{E'}^{\lambda}_{1,2}(x, y) \cdot \exp\left[i\frac{2\pi}{\lambda}\frac{(x_1 - x)^2 + (y_1 - y)^2}{2{l_\lambda}'}\right]dxdy$$

$$\propto F\{P_{1,2}(x, y)\}|_{u=\frac{x_1}{\lambda f}, v=\frac{y_1}{\lambda f}} \qquad (S27)$$

where (x_1, y_1) is the 2D coordinate at the image plane, (u, v) is the spatial frequency of the metasurface-modulated light field along the x and y axes, and F denotes the Fourier transform. Equation S27 indicates that the metasurfaces' PSF, $PSF_{1,2}^{\lambda}$, remains consistent across a broad wavelength range.

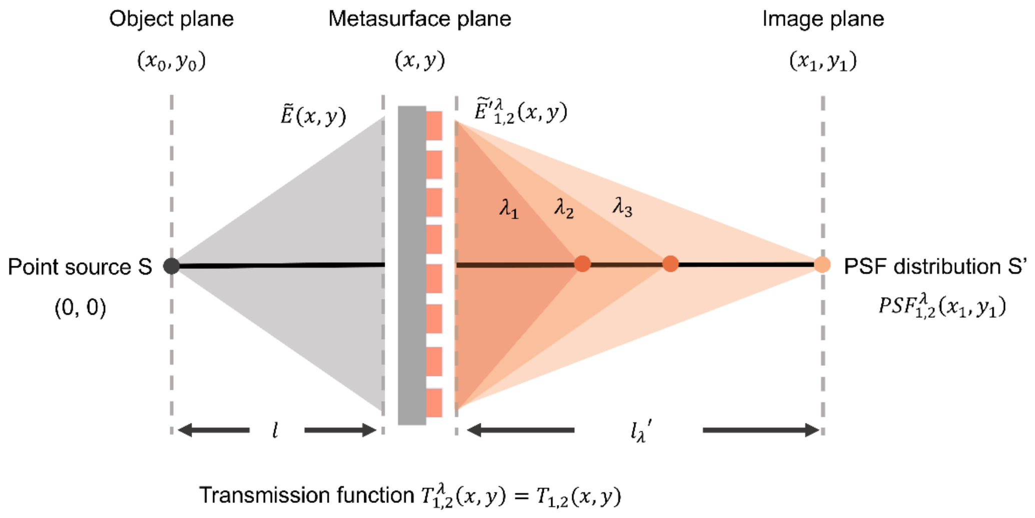

Fig. S7: Schematic representation of the metasurface differentiator under different illumination wavelengths. A point source S is located at the coordinate origin $(0, 0)$ of the object plane, with a distance l from the metasurface plane. The metasurface results in a corresponding PSF distribution S' at the image plane, with a distance l_{λ}' from the metasurface plane.

Section S9. Demonstration of other advanced all-optical computing functionalities

The PSF-engineering approach demonstrated in this work is indeed a general methodology and can be employed to realize a wide range of linear mathematical operations. As a proof of concept, we numerically demonstrate a metasurface device that is capable of performing denoising and edge-enhancing operations under LCP and RCP illumination, respectively. The device has a diameter of D = 3.000 mm and numerical aperture of NA = 0.3, corresponding to a focal length of f_0 = 4.770 mm.

Under LCP illumination, the simulated PSF intensity and phase profiles are displayed in Fig. S8a. A line pair with resolution of 71.8 lp/mm, which equals to the resolution of element 2 of line pair group #6 in the 1951 USAF resolution test chart, can be clearly resolved in the presence of salt-and-pepper noise (Fig. S8b), confirming the device's denoising functionality and its ability to maintain high imaging quality.

Under RCP illumination, the simulated PSF intensity and phase profiles are displayed in Fig. S8c. A line pair with resolution of 71.8 lp/mm, which equals to the resolution of element 2 of line pair group #6 in the 1951 USAF resolution test chart, can be clearly resolved (Fig. S8b). The associated cross-sectional plots along the white dashed line (Fig. S8b, right panel) demonstrate the edge-enhancing functionality.

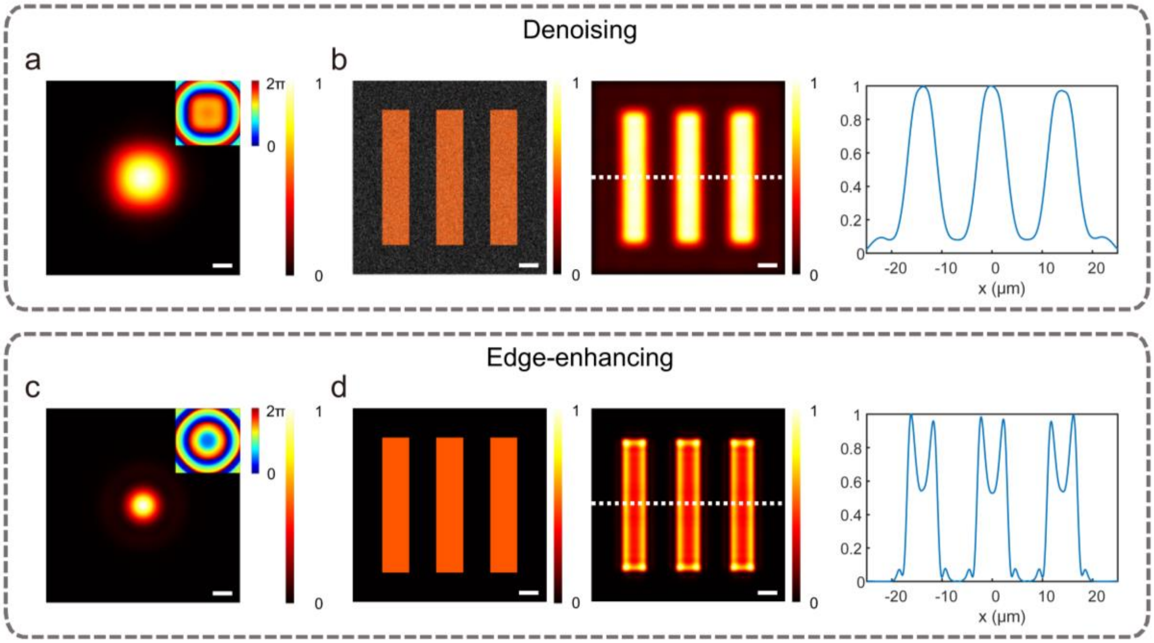

Fig. S8: Numerical evaluation of the performance of the denoising and edge-enhancing operations. (a) Simulated complex-valued PSF distributions corresponding to the denoising operation under LCP illumination. Scale bar: 1 μm. (b) Target object with salt-and-pepper noise (left panel), simulated imaging results (middle panel), and the associated cross-sectional plots along the white dashed line (right panel) under LCP illumination. Scale bar: 5 μm. (c) Simulated complex-valued PSF distributions corresponding to the edge-enhancing operation under RCP illumination. Scale bar: 1 μm. (d) Target object (left panel), simulated imaging results (middle panel), and the associated cross-sectional plots along the white dashed line (right panel) under RCP illumination. Scale bar: 5 μm.

References

1. Liu, Z. *et al*. Metasurface-enabled augmented reality display: a review. *Advanced Photonics* **5**, 034001 (2023).
